# Supplementary figures and images for: CDK1-mediated phosphorylation of LDHA fuels mitosis through LDHB-dependent lactate oxidation
Source: EMBO Rep. 2025 Sep 12;26(20):4923–49. doi: 10.1038/s44319-025-00573-8 (PMC12550033; doi:10.1038/s44319-025-00573-8)

**Fig. 1A**

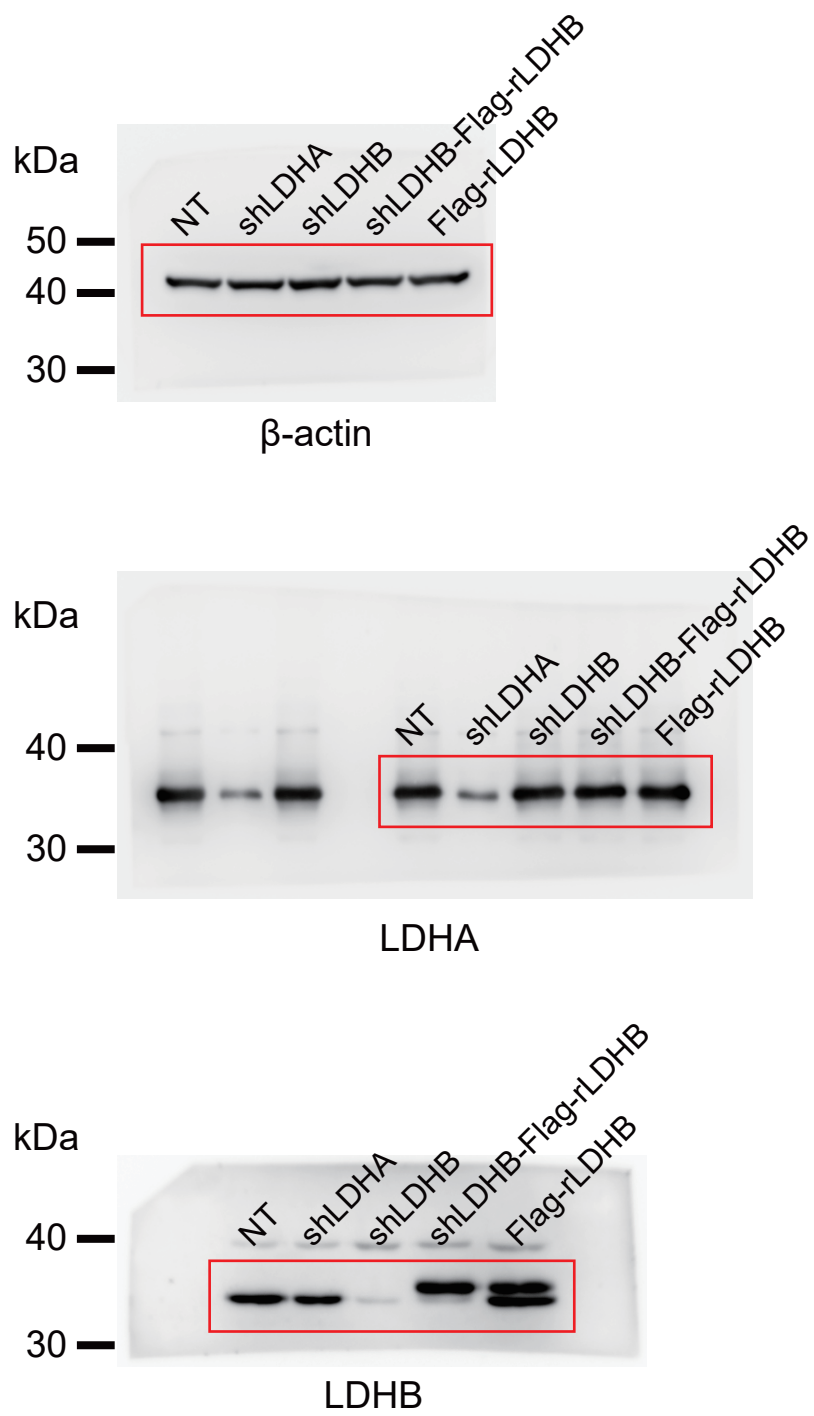

Supplement: Supplementary file 2 — Source data Fig. 1 [file 44319_2025_573_MOESM2_ESM.zip › Figure 1 Source Data/1A/Fig. 1A-β actin-LDHA-LDHB.pdf]

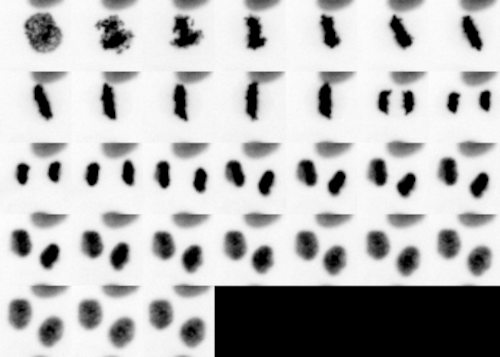

Supplement: Supplementary file 2 — Source data Fig. 1 [file 44319_2025_573_MOESM2_ESM.zip › Figure 1 Source Data/1A/Flag-LDHB.tif]

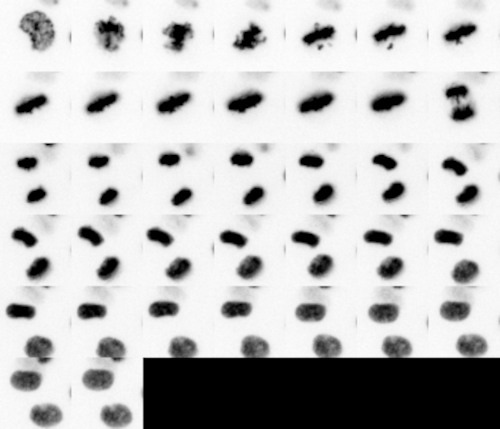

Supplement: Supplementary file 2 — Source data Fig. 1 [file 44319_2025_573_MOESM2_ESM.zip › Figure 1 Source Data/1A/LDHA KD.tif]

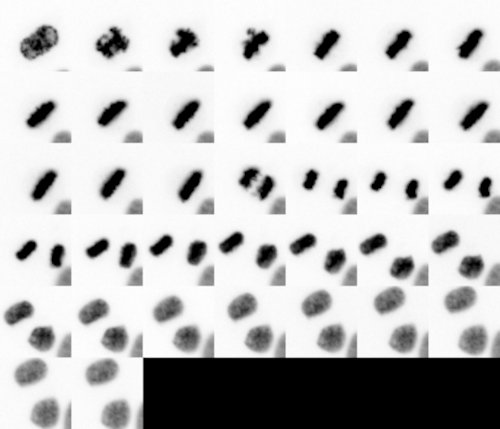

Supplement: Supplementary file 2 — Source data Fig. 1 [file 44319_2025_573_MOESM2_ESM.zip › Figure 1 Source Data/1A/LDHB KD + Flag-rLDHB.tif]

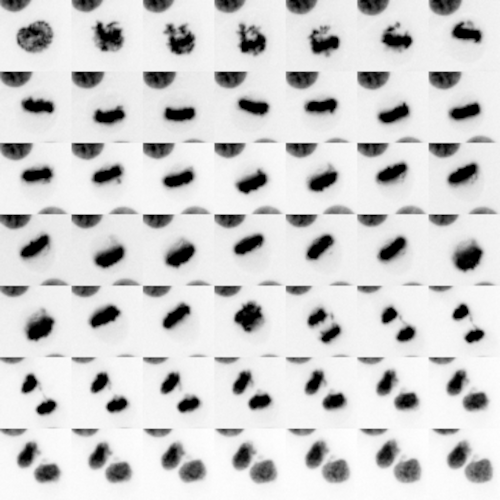

Supplement: Supplementary file 2 — Source data Fig. 1 [file 44319_2025_573_MOESM2_ESM.zip › Figure 1 Source Data/1A/LDHB KD.tif]

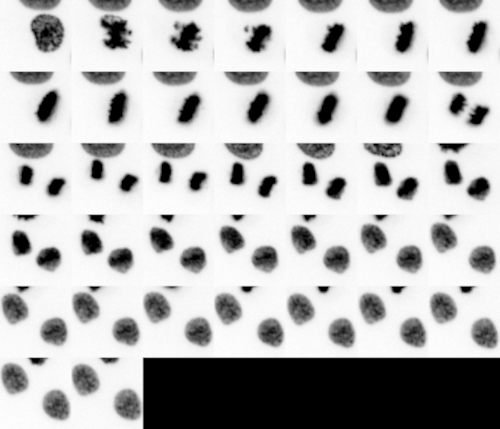

Supplement: Supplementary file 2 — Source data Fig. 1 [file 44319_2025_573_MOESM2_ESM.zip › Figure 1 Source Data/1A/NT.tif]

Fig. 1C

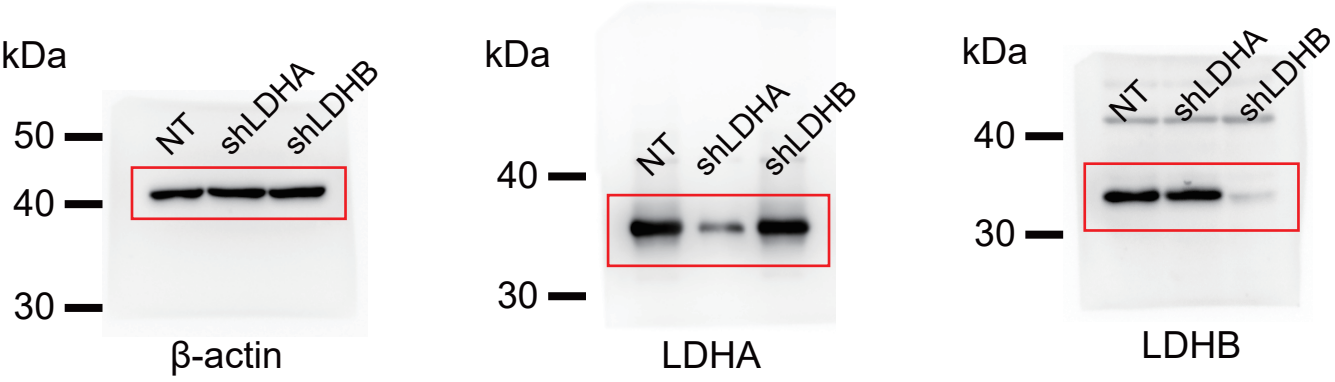

Supplement: Supplementary file 2 — Source data Fig. 1 [file 44319_2025_573_MOESM2_ESM.zip › Figure 1 Source Data/1C/Fig. 1C-β actin-LDHA-LDHB.pdf]

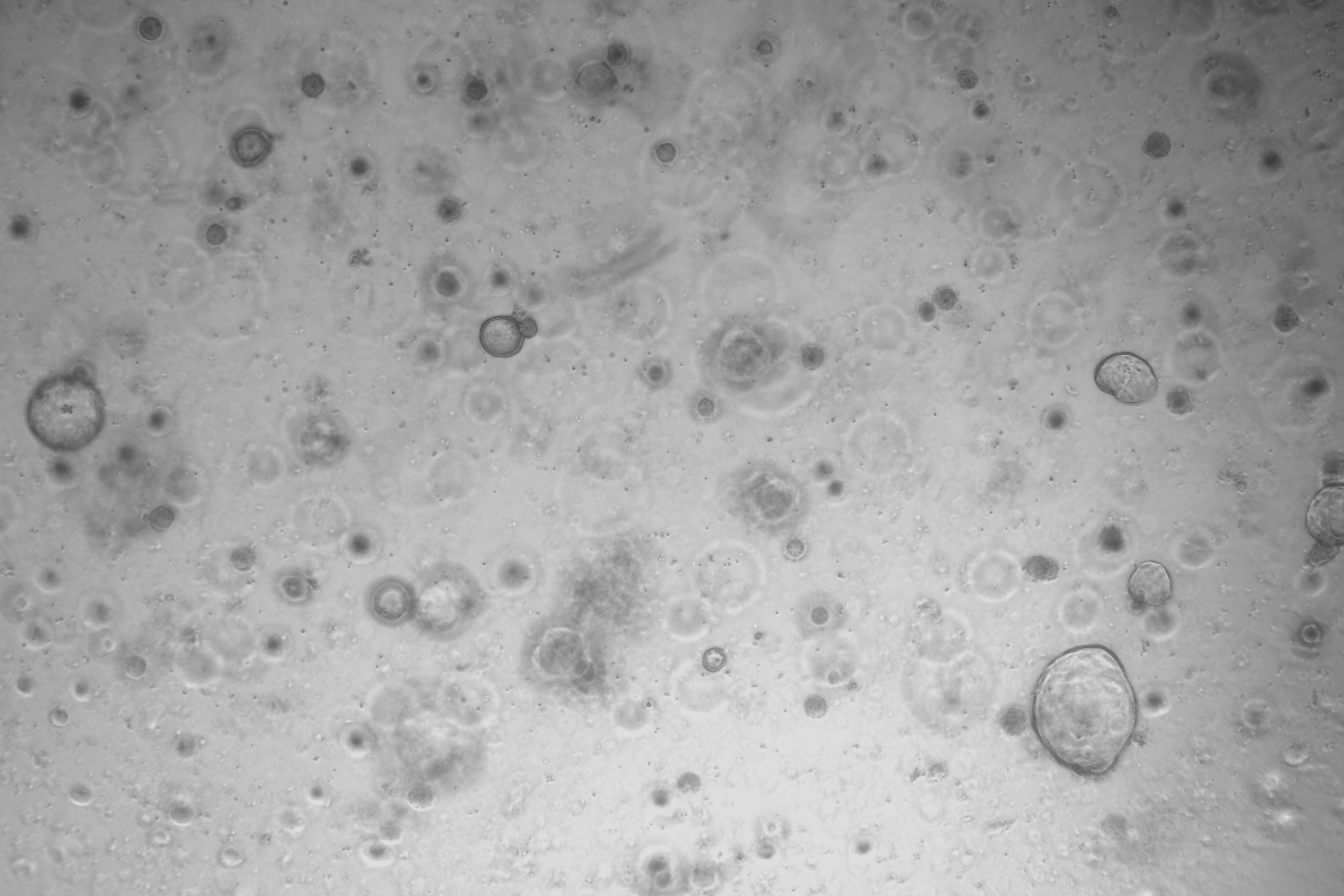

Supplement: Supplementary file 2 — Source data Fig. 1 [file 44319_2025_573_MOESM2_ESM.zip › Figure 1 Source Data/1C/LDHA KD.tif]

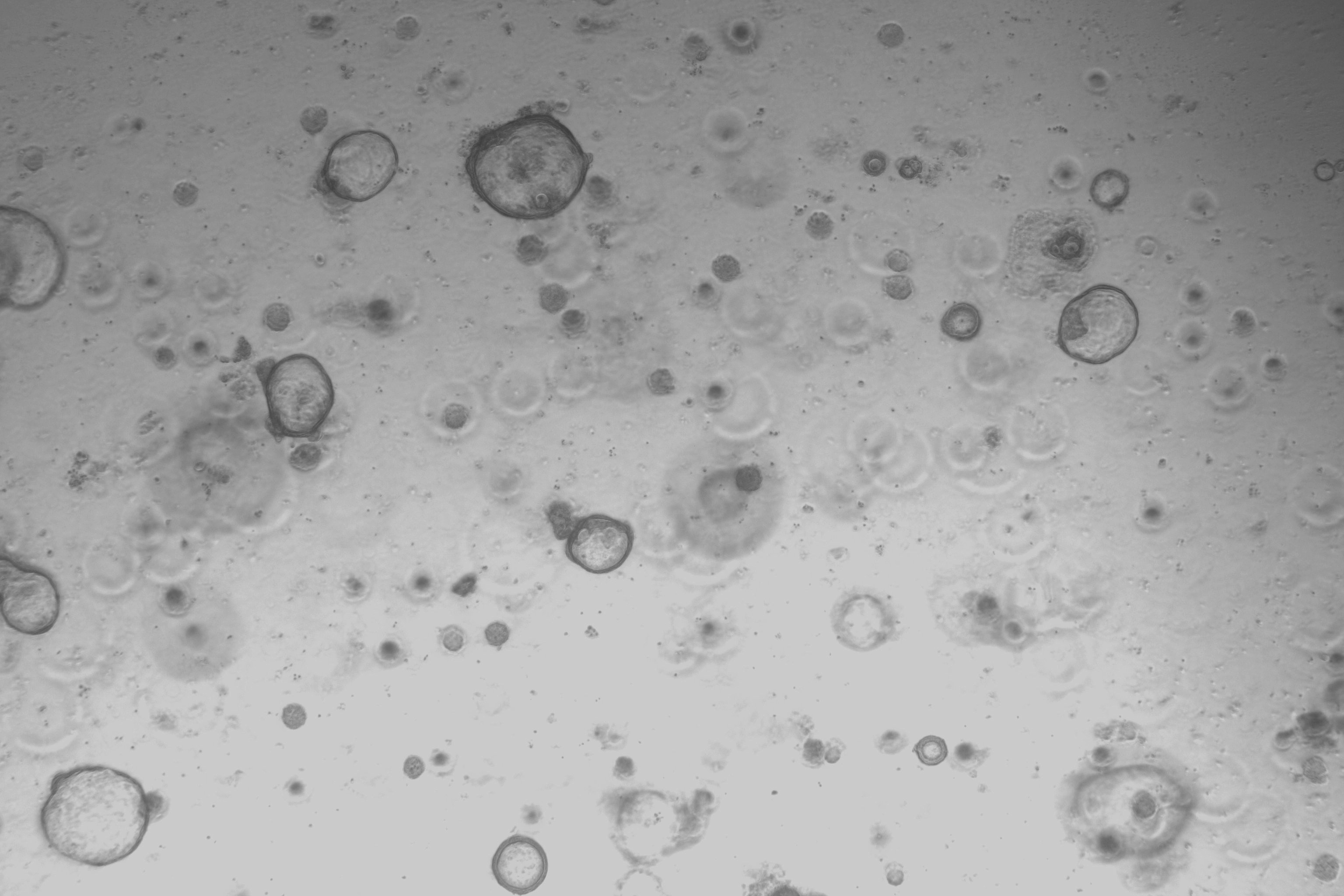

Supplement: Supplementary file 2 — Source data Fig. 1 [file 44319_2025_573_MOESM2_ESM.zip › Figure 1 Source Data/1C/LDHB KD.tif]

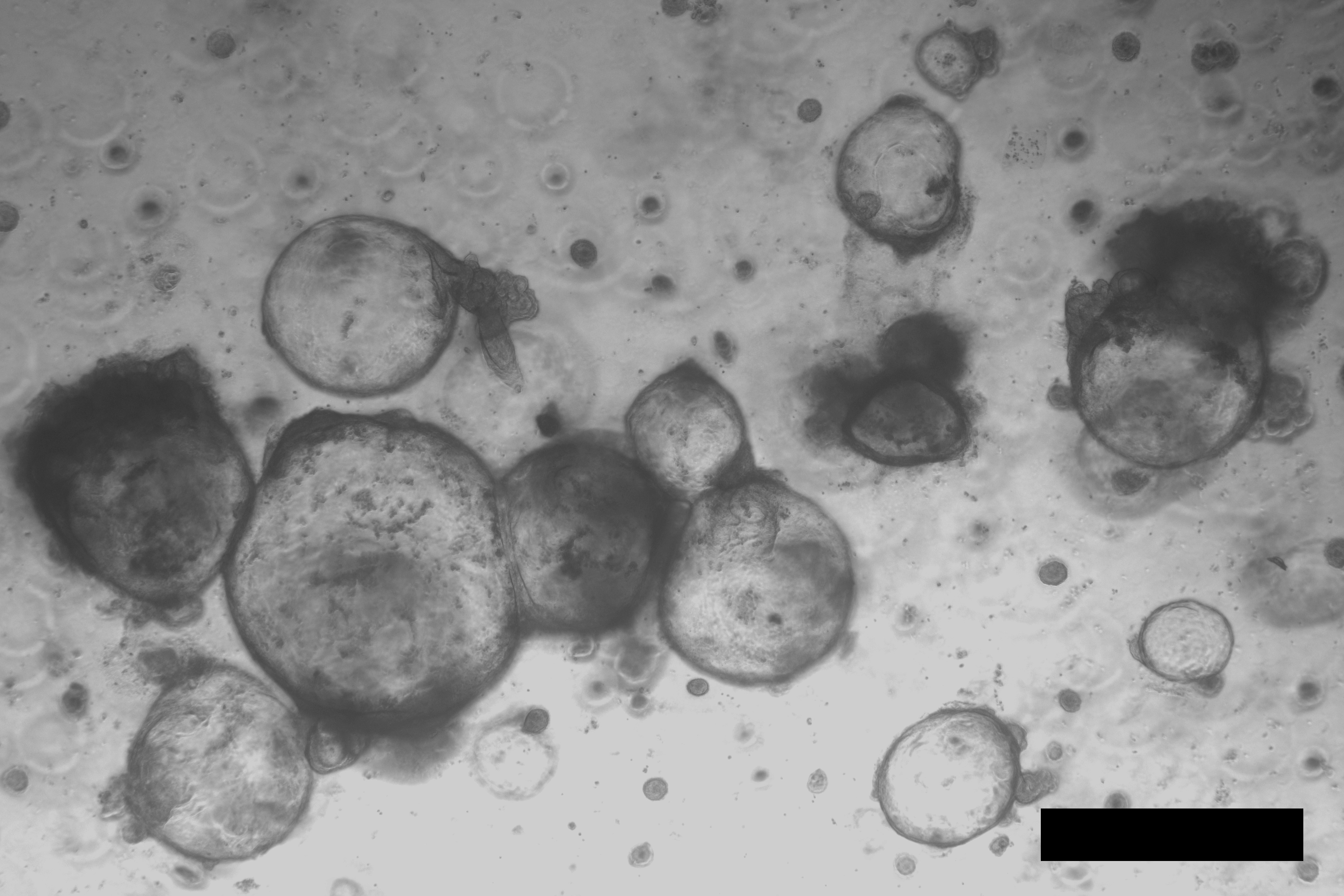

Supplement: Supplementary file 2 — Source data Fig. 1 [file 44319_2025_573_MOESM2_ESM.zip › Figure 1 Source Data/1C/NT-scale bar 200 μm.tif]

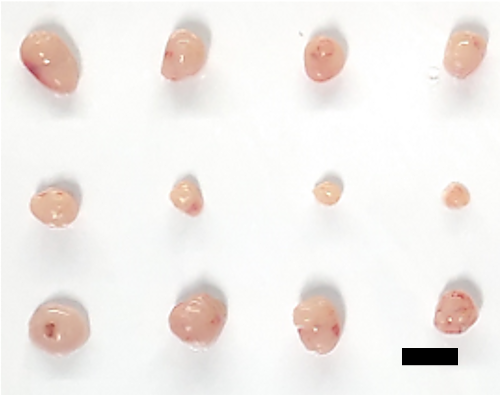

Supplement: Supplementary file 2 — Source data Fig. 1 [file 44319_2025_573_MOESM2_ESM.zip › Figure 1 Source Data/1D/Fig. 1D mouse-tumor- scale bar 1cm.tif]

Fig. 1F

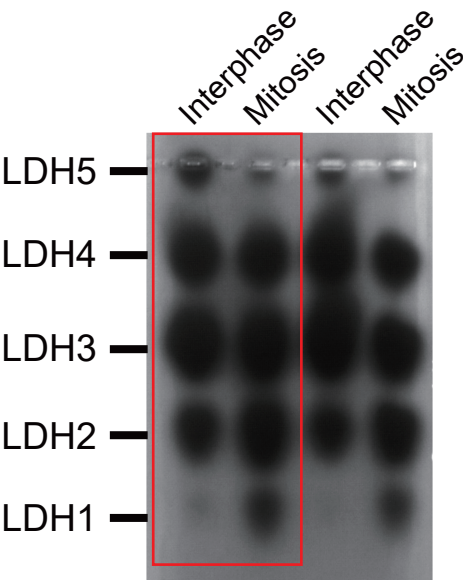

Supplement: Supplementary file 2 — Source data Fig. 1 [file 44319_2025_573_MOESM2_ESM.zip › Figure 1 Source Data/1F/Fig. 1F-LDH.pdf]

Fig. 1G

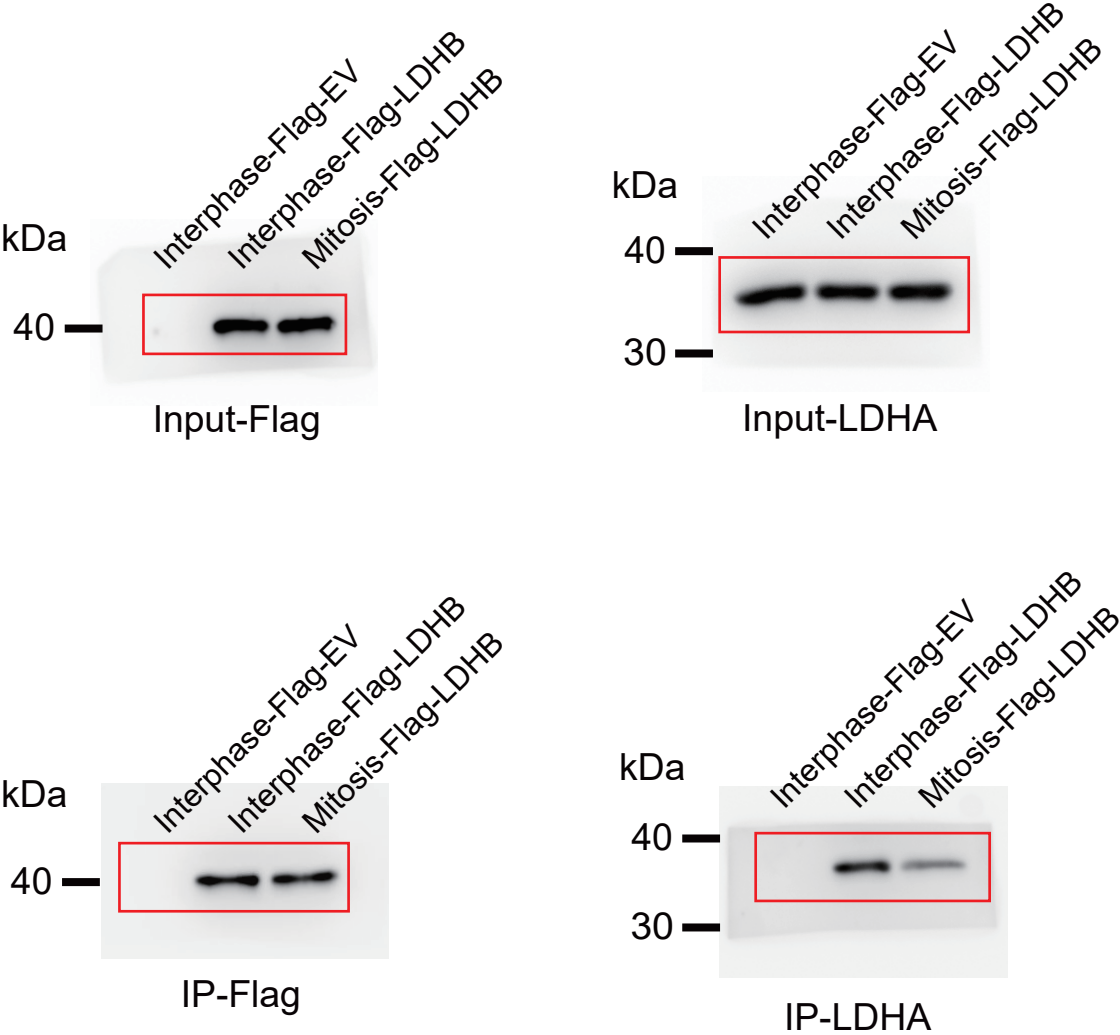

Supplement: Supplementary file 2 — Source data Fig. 1 [file 44319_2025_573_MOESM2_ESM.zip › Figure 1 Source Data/1G/Fig. 1G-Co IP-Flag-LDHA.pdf]

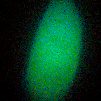

Supplement: Supplementary file 3 — Source data Fig. 2 [file 44319_2025_573_MOESM3_ESM.zip › Figure 2 Source Data/2C/Interphase-cpYFP-Color Ratio.tif]

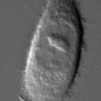

Supplement: Supplementary file 3 — Source data Fig. 2 [file 44319_2025_573_MOESM3_ESM.zip › Figure 2 Source Data/2C/Interphase-cpYFP-DIC.tif]

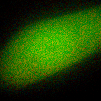

Supplement: Supplementary file 3 — Source data Fig. 2 [file 44319_2025_573_MOESM3_ESM.zip › Figure 2 Source Data/2C/Interphase-SoNar-Color Ratio.tif]

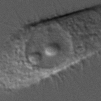

Supplement: Supplementary file 3 — Source data Fig. 2 [file 44319_2025_573_MOESM3_ESM.zip › Figure 2 Source Data/2C/Interphase-SoNar-DIC.tif]

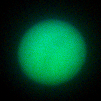

Supplement: Supplementary file 3 — Source data Fig. 2 [file 44319_2025_573_MOESM3_ESM.zip › Figure 2 Source Data/2C/Mitosis shLDHB-cpYFP-Color Ratio.tif]

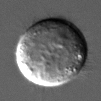

Supplement: Supplementary file 3 — Source data Fig. 2 [file 44319_2025_573_MOESM3_ESM.zip › Figure 2 Source Data/2C/Mitosis shLDHB-cpYFP-DIC.tif]

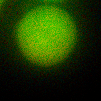

Supplement: Supplementary file 3 — Source data Fig. 2 [file 44319_2025_573_MOESM3_ESM.zip › Figure 2 Source Data/2C/Mitosis shLDHB-SoNar-Color Ratio.tif]

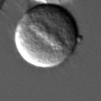

Supplement: Supplementary file 3 — Source data Fig. 2 [file 44319_2025_573_MOESM3_ESM.zip › Figure 2 Source Data/2C/Mitosis shLDHB-SoNar-DIC.tif]

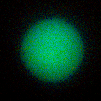

Supplement: Supplementary file 3 — Source data Fig. 2 [file 44319_2025_573_MOESM3_ESM.zip › Figure 2 Source Data/2C/Mitosis-cpYFP-Color Ratio.tif]

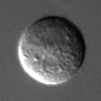

Supplement: Supplementary file 3 — Source data Fig. 2 [file 44319_2025_573_MOESM3_ESM.zip › Figure 2 Source Data/2C/Mitosis-cpYFP-DIC.tif]

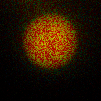

Supplement: Supplementary file 3 — Source data Fig. 2 [file 44319_2025_573_MOESM3_ESM.zip › Figure 2 Source Data/2C/Mitosis-SoNar-Color Ratio.tif]

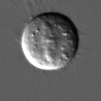

Supplement: Supplementary file 3 — Source data Fig. 2 [file 44319_2025_573_MOESM3_ESM.zip › Figure 2 Source Data/2C/Mitosis-SoNar-DIC.tif]

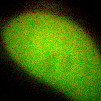

Supplement: Supplementary file 4 — Source data Fig. 3 [file 44319_2025_573_MOESM4_ESM.zip › Figure 3 Source Data/3B/Fila C-interphase-Color Ratio.tif]

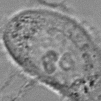

Supplement: Supplementary file 4 — Source data Fig. 3 [file 44319_2025_573_MOESM4_ESM.zip › Figure 3 Source Data/3B/Fila C-interphase-DIC.tif]

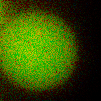

Supplement: Supplementary file 4 — Source data Fig. 3 [file 44319_2025_573_MOESM4_ESM.zip › Figure 3 Source Data/3B/Fila C-mitosis-Color Ratio.tif]

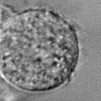

Supplement: Supplementary file 4 — Source data Fig. 3 [file 44319_2025_573_MOESM4_ESM.zip › Figure 3 Source Data/3B/Fila C-mitosis-DIC.tif]

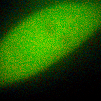

Supplement: Supplementary file 4 — Source data Fig. 3 [file 44319_2025_573_MOESM4_ESM.zip › Figure 3 Source Data/3B/Fila-interphase-Color Ratio.tif]

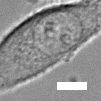

Supplement: Supplementary file 4 — Source data Fig. 3 [file 44319_2025_573_MOESM4_ESM.zip › Figure 3 Source Data/3B/Fila-interphase-DIC.tif]

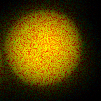

Supplement: Supplementary file 4 — Source data Fig. 3 [file 44319_2025_573_MOESM4_ESM.zip › Figure 3 Source Data/3B/Fila-mitosis-Color Ratio.tif]

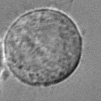

Supplement: Supplementary file 4 — Source data Fig. 3 [file 44319_2025_573_MOESM4_ESM.zip › Figure 3 Source Data/3B/Fila-mitosis-DIC.tif]

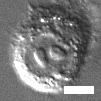

Supplement: Supplementary file 4 — Source data Fig. 3 [file 44319_2025_573_MOESM4_ESM.zip › Figure 3 Source Data/3C/FiLa-Interphase-DIC.tif]

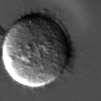

Supplement: Supplementary file 4 — Source data Fig. 3 [file 44319_2025_573_MOESM4_ESM.zip › Figure 3 Source Data/3C/FiLa-Mitosis-DIC.tif]

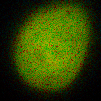

Supplement: Supplementary file 4 — Source data Fig. 3 [file 44319_2025_573_MOESM4_ESM.zip › Figure 3 Source Data/3C/Interphase-0 min.tif]

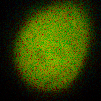

Supplement: Supplementary file 4 — Source data Fig. 3 [file 44319_2025_573_MOESM4_ESM.zip › Figure 3 Source Data/3C/Interphase-2 min.tif]

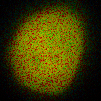

Supplement: Supplementary file 4 — Source data Fig. 3 [file 44319_2025_573_MOESM4_ESM.zip › Figure 3 Source Data/3C/Interphase-3 min.tif]

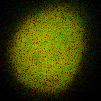

Supplement: Supplementary file 4 — Source data Fig. 3 [file 44319_2025_573_MOESM4_ESM.zip › Figure 3 Source Data/3C/Interphase-4 min.tif]

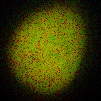

Supplement: Supplementary file 4 — Source data Fig. 3 [file 44319_2025_573_MOESM4_ESM.zip › Figure 3 Source Data/3C/Interphase-5 min.tif]

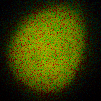

Supplement: Supplementary file 4 — Source data Fig. 3 [file 44319_2025_573_MOESM4_ESM.zip › Figure 3 Source Data/3C/Interphase-6 min.tif]

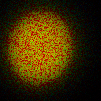

Supplement: Supplementary file 4 — Source data Fig. 3 [file 44319_2025_573_MOESM4_ESM.zip › Figure 3 Source Data/3C/Mitosis-0 min.tif]

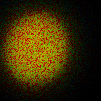

Supplement: Supplementary file 4 — Source data Fig. 3 [file 44319_2025_573_MOESM4_ESM.zip › Figure 3 Source Data/3C/Mitosis-2 min.tif]

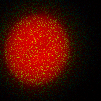

Supplement: Supplementary file 4 — Source data Fig. 3 [file 44319_2025_573_MOESM4_ESM.zip › Figure 3 Source Data/3C/Mitosis-3 min.tif]

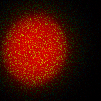

Supplement: Supplementary file 4 — Source data Fig. 3 [file 44319_2025_573_MOESM4_ESM.zip › Figure 3 Source Data/3C/Mitosis-4 min.tif]

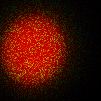

Supplement: Supplementary file 4 — Source data Fig. 3 [file 44319_2025_573_MOESM4_ESM.zip › Figure 3 Source Data/3C/Mitosis-5min.tif]

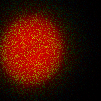

Supplement: Supplementary file 4 — Source data Fig. 3 [file 44319_2025_573_MOESM4_ESM.zip › Figure 3 Source Data/3C/Mitosis-6 min.tif]

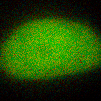

Supplement: Supplementary file 4 — Source data Fig. 3 [file 44319_2025_573_MOESM4_ESM.zip › Figure 3 Source Data/3D/Interphase-0 min.tif]

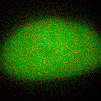

Supplement: Supplementary file 4 — Source data Fig. 3 [file 44319_2025_573_MOESM4_ESM.zip › Figure 3 Source Data/3D/Interphase-2 min.tif]

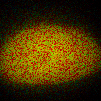

Supplement: Supplementary file 4 — Source data Fig. 3 [file 44319_2025_573_MOESM4_ESM.zip › Figure 3 Source Data/3D/Interphase-3 min.tif]

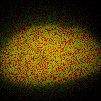

Supplement: Supplementary file 4 — Source data Fig. 3 [file 44319_2025_573_MOESM4_ESM.zip › Figure 3 Source Data/3D/Interphase-4 min.tif]

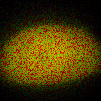

Supplement: Supplementary file 4 — Source data Fig. 3 [file 44319_2025_573_MOESM4_ESM.zip › Figure 3 Source Data/3D/Interphase-5 min.tif]

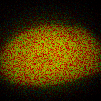

Supplement: Supplementary file 4 — Source data Fig. 3 [file 44319_2025_573_MOESM4_ESM.zip › Figure 3 Source Data/3D/Interphase-6 min.tif]

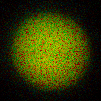

Supplement: Supplementary file 4 — Source data Fig. 3 [file 44319_2025_573_MOESM4_ESM.zip › Figure 3 Source Data/3D/Mitosis-0 min.tif]

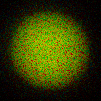

Supplement: Supplementary file 4 — Source data Fig. 3 [file 44319_2025_573_MOESM4_ESM.zip › Figure 3 Source Data/3D/Mitosis-2 min.tif]

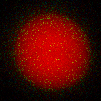

Supplement: Supplementary file 4 — Source data Fig. 3 [file 44319_2025_573_MOESM4_ESM.zip › Figure 3 Source Data/3D/Mitosis-3 min.tif]

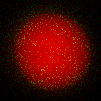

Supplement: Supplementary file 4 — Source data Fig. 3 [file 44319_2025_573_MOESM4_ESM.zip › Figure 3 Source Data/3D/Mitosis-4 min.tif]

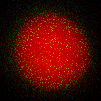

Supplement: Supplementary file 4 — Source data Fig. 3 [file 44319_2025_573_MOESM4_ESM.zip › Figure 3 Source Data/3D/Mitosis-5 min.tif]

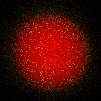

Supplement: Supplementary file 4 — Source data Fig. 3 [file 44319_2025_573_MOESM4_ESM.zip › Figure 3 Source Data/3D/Mitosis-6 min.tif]

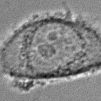

Supplement: Supplementary file 4 — Source data Fig. 3 [file 44319_2025_573_MOESM4_ESM.zip › Figure 3 Source Data/3D/SoNar-Interphase-DIC.tif]

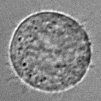

Supplement: Supplementary file 4 — Source data Fig. 3 [file 44319_2025_573_MOESM4_ESM.zip › Figure 3 Source Data/3D/SoNar-Mitosis-DIC.tif]

Fig. 3E

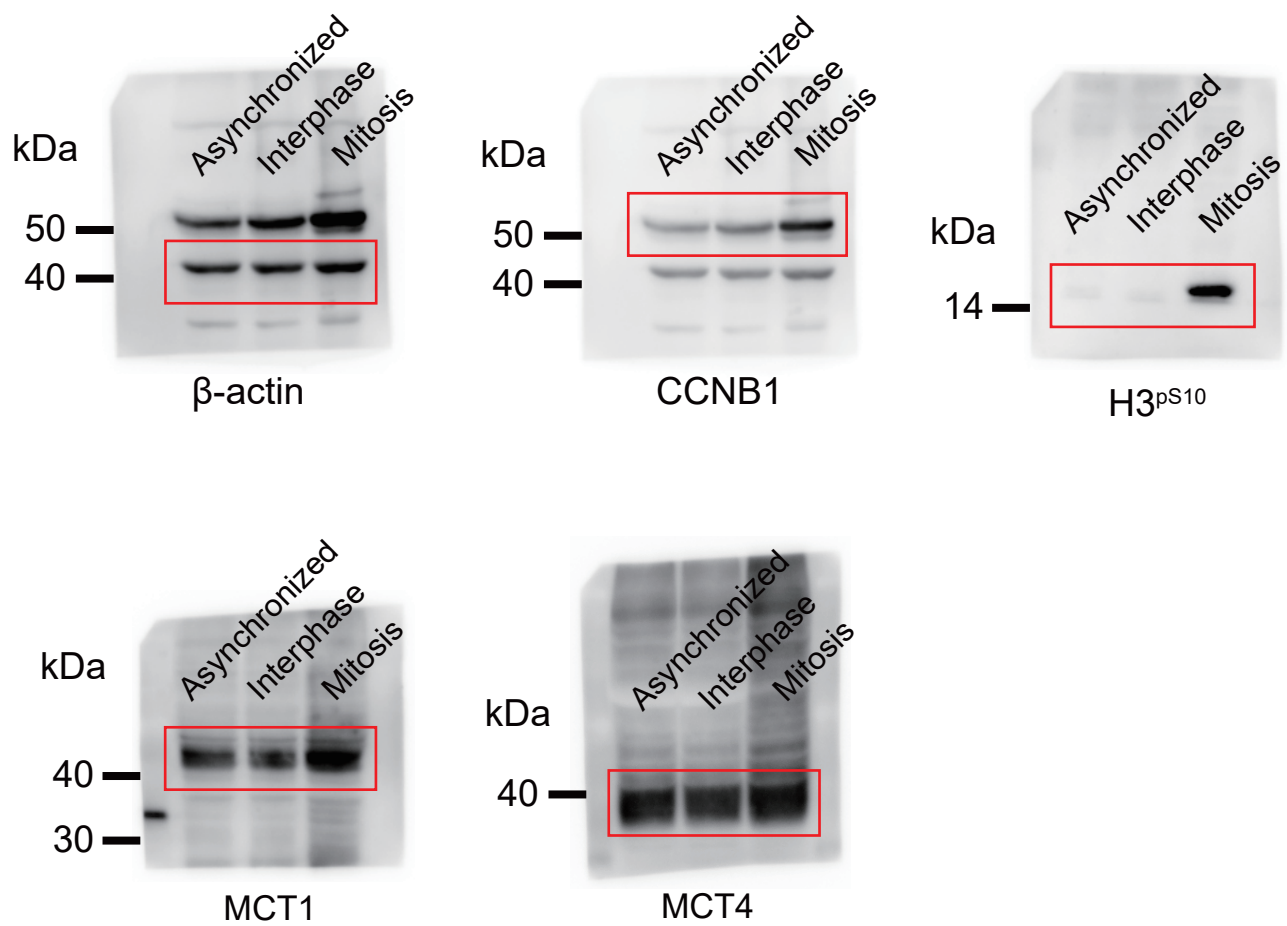

Supplement: Supplementary file 4 — Source data Fig. 3 [file 44319_2025_573_MOESM4_ESM.zip › Figure 3 Source Data/3E/Fig.3E-β actin-CCNB1-MCT1-MCT4.pdf]

Fig. 3G

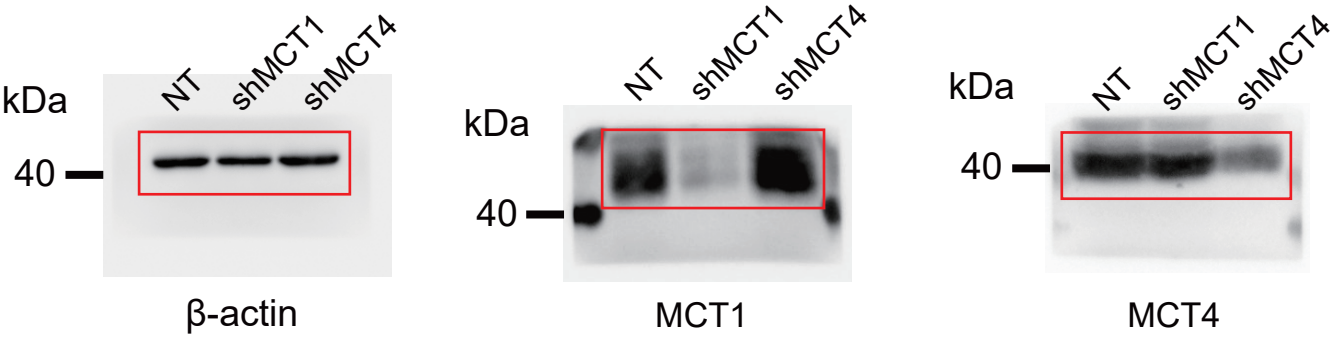

Supplement: Supplementary file 4 — Source data Fig. 3 [file 44319_2025_573_MOESM4_ESM.zip › Figure 3 Source Data/3G/Fig.3G-β actin-MCT1-MCT4.pdf]

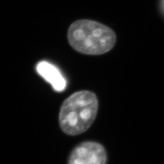

Supplement: Supplementary file 5 — Source data Fig. 4 [file 44319_2025_573_MOESM5_ESM.zip › Figure 4 Source Data/4C/DAPI-001.tif]

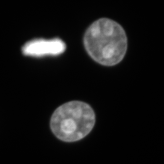

Supplement: Supplementary file 5 — Source data Fig. 4 [file 44319_2025_573_MOESM5_ESM.zip › Figure 4 Source Data/4C/DAPI-002.tif]

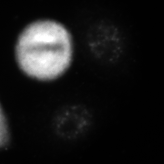

Supplement: Supplementary file 5 — Source data Fig. 4 [file 44319_2025_573_MOESM5_ESM.zip › Figure 4 Source Data/4C/LDHA pT18.tif]

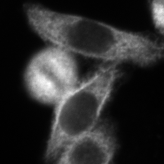

Supplement: Supplementary file 5 — Source data Fig. 4 [file 44319_2025_573_MOESM5_ESM.zip › Figure 4 Source Data/4C/LDHA.tif]

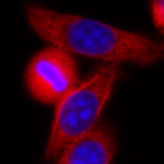

Supplement: Supplementary file 5 — Source data Fig. 4 [file 44319_2025_573_MOESM5_ESM.zip › Figure 4 Source Data/4C/Merge-1.tif]

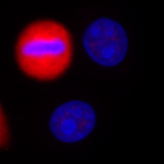

Supplement: Supplementary file 5 — Source data Fig. 4 [file 44319_2025_573_MOESM5_ESM.zip › Figure 4 Source Data/4C/Merge-2.tif]

Fig. 4E

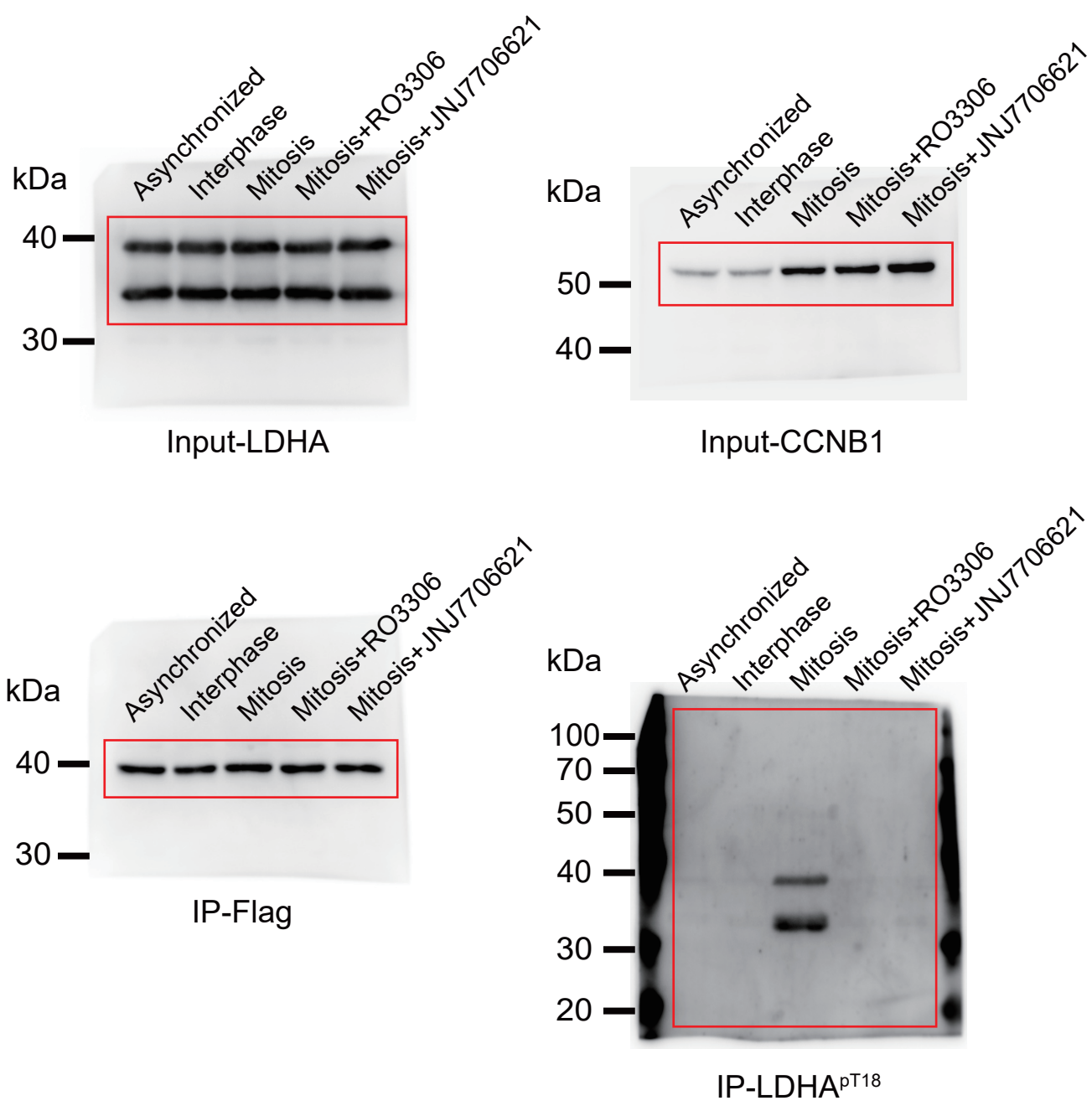

Supplement: Supplementary file 5 — Source data Fig. 4 [file 44319_2025_573_MOESM5_ESM.zip › Figure 4 Source Data/4E/Fig. 4E-Co IP-CCNB1-LDHA-Flag-LDHA pT18.pdf]

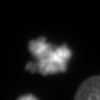

Supplement: Supplementary file 5 — Source data Fig. 4 [file 44319_2025_573_MOESM5_ESM.zip › Figure 4 Source Data/4F/BI2536-DAPI.tif]

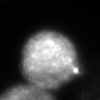

Supplement: Supplementary file 5 — Source data Fig. 4 [file 44319_2025_573_MOESM5_ESM.zip › Figure 4 Source Data/4F/BI2536-LDHA pT18.tif]

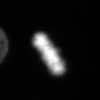

Supplement: Supplementary file 5 — Source data Fig. 4 [file 44319_2025_573_MOESM5_ESM.zip › Figure 4 Source Data/4F/DMSO-DAPI.tif]

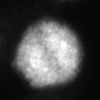

Supplement: Supplementary file 5 — Source data Fig. 4 [file 44319_2025_573_MOESM5_ESM.zip › Figure 4 Source Data/4F/DMSO-LDHA pT18.tif]

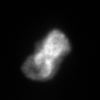

Supplement: Supplementary file 5 — Source data Fig. 4 [file 44319_2025_573_MOESM5_ESM.zip › Figure 4 Source Data/4F/JNJ7706621-DAPI.tif]

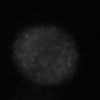

Supplement: Supplementary file 5 — Source data Fig. 4 [file 44319_2025_573_MOESM5_ESM.zip › Figure 4 Source Data/4F/JNJ7706621-LDHA pT18.tif]

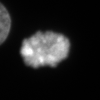

Supplement: Supplementary file 5 — Source data Fig. 4 [file 44319_2025_573_MOESM5_ESM.zip › Figure 4 Source Data/4F/MLN8237-DAPI.tif]

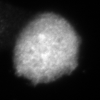

Supplement: Supplementary file 5 — Source data Fig. 4 [file 44319_2025_573_MOESM5_ESM.zip › Figure 4 Source Data/4F/MLN8237-LDHA pT18.tif]

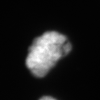

Supplement: Supplementary file 5 — Source data Fig. 4 [file 44319_2025_573_MOESM5_ESM.zip › Figure 4 Source Data/4F/RO3306-DAPI.tif]

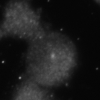

Supplement: Supplementary file 5 — Source data Fig. 4 [file 44319_2025_573_MOESM5_ESM.zip › Figure 4 Source Data/4F/RO3306-LDHA pT18.tif]

Fig. 4G

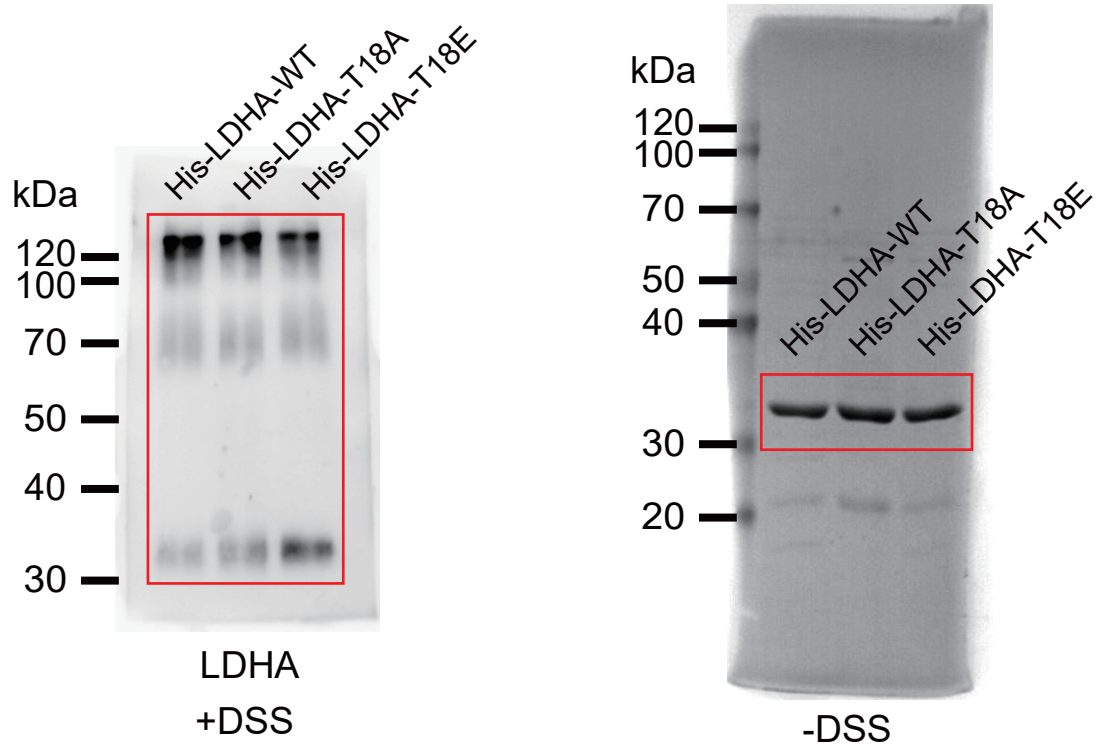

Supplement: Supplementary file 5 — Source data Fig. 4 [file 44319_2025_573_MOESM5_ESM.zip › Figure 4 Source Data/4G/Fig.4G-DSS-LDHA.pdf]

Fig. 4H

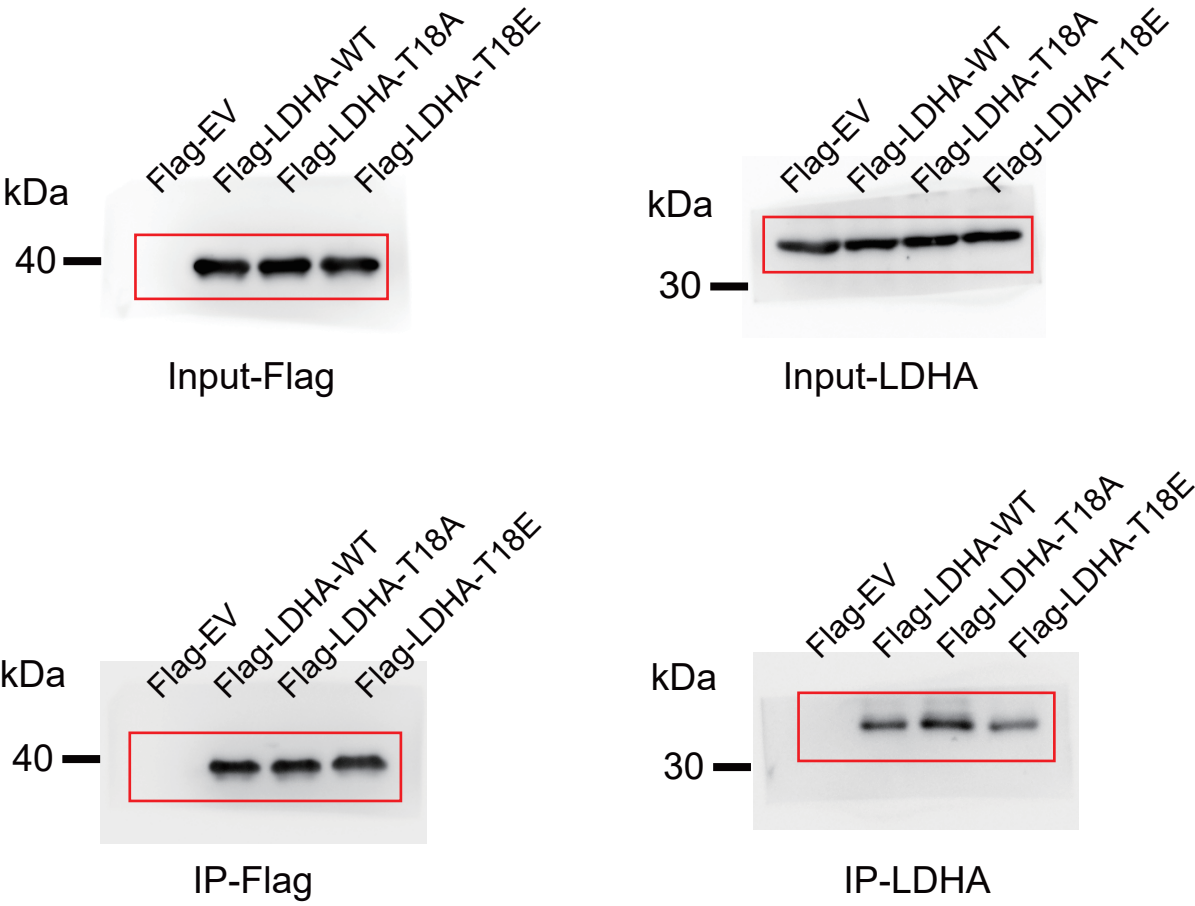

Supplement: Supplementary file 5 — Source data Fig. 4 [file 44319_2025_573_MOESM5_ESM.zip › Figure 4 Source Data/4H/Fig.4H-CoIP-Flag-LDHA.pdf]

Fig. 4I

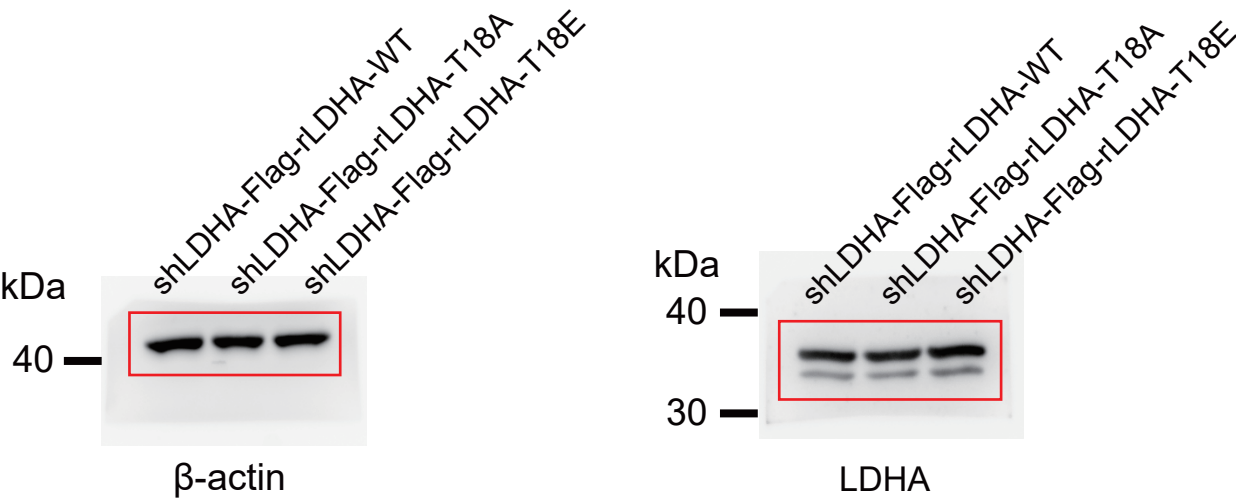

Supplement: Supplementary file 5 — Source data Fig. 4 [file 44319_2025_573_MOESM5_ESM.zip › Figure 4 Source Data/4I/Fig.4I.pdf]

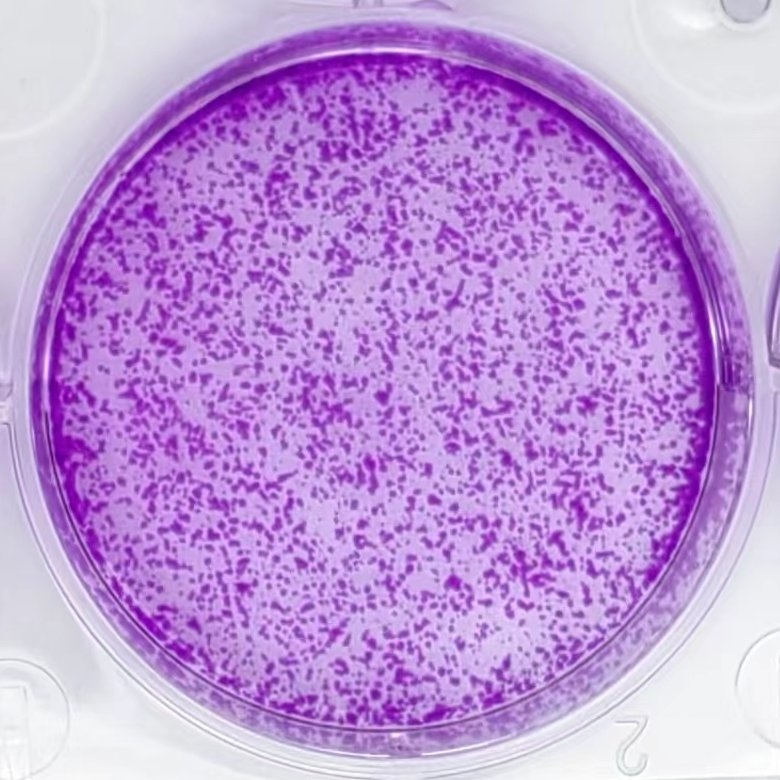

Supplement: Supplementary file 6 — Source data Fig. 5 [file 44319_2025_573_MOESM6_ESM.zip › Figure 5 Source Data/5A/Gipz-shLDHA-LDHA-T18A.tif]

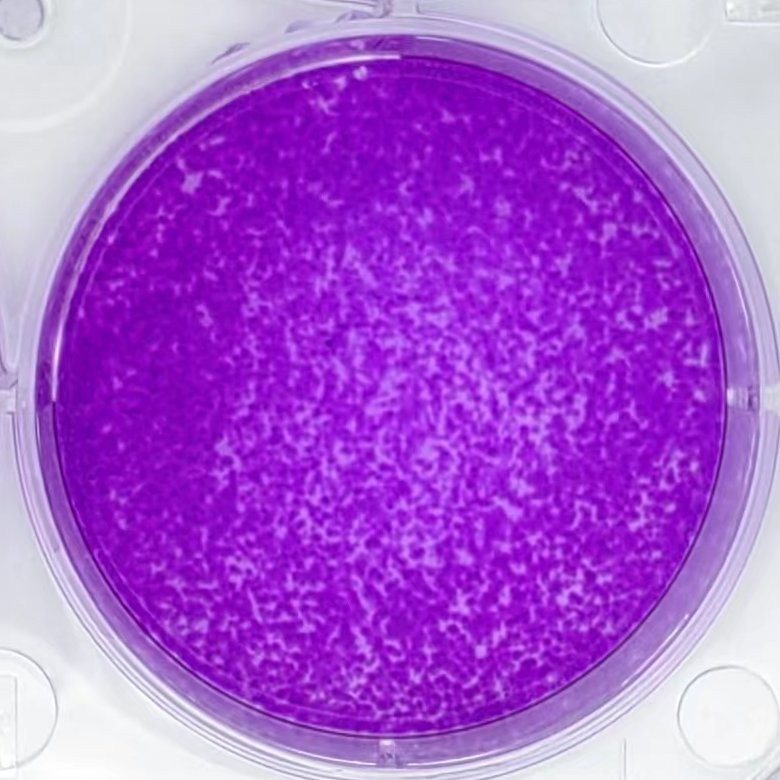

Supplement: Supplementary file 6 — Source data Fig. 5 [file 44319_2025_573_MOESM6_ESM.zip › Figure 5 Source Data/5A/Gipz-shLDHA-LDHA-WT.tif]

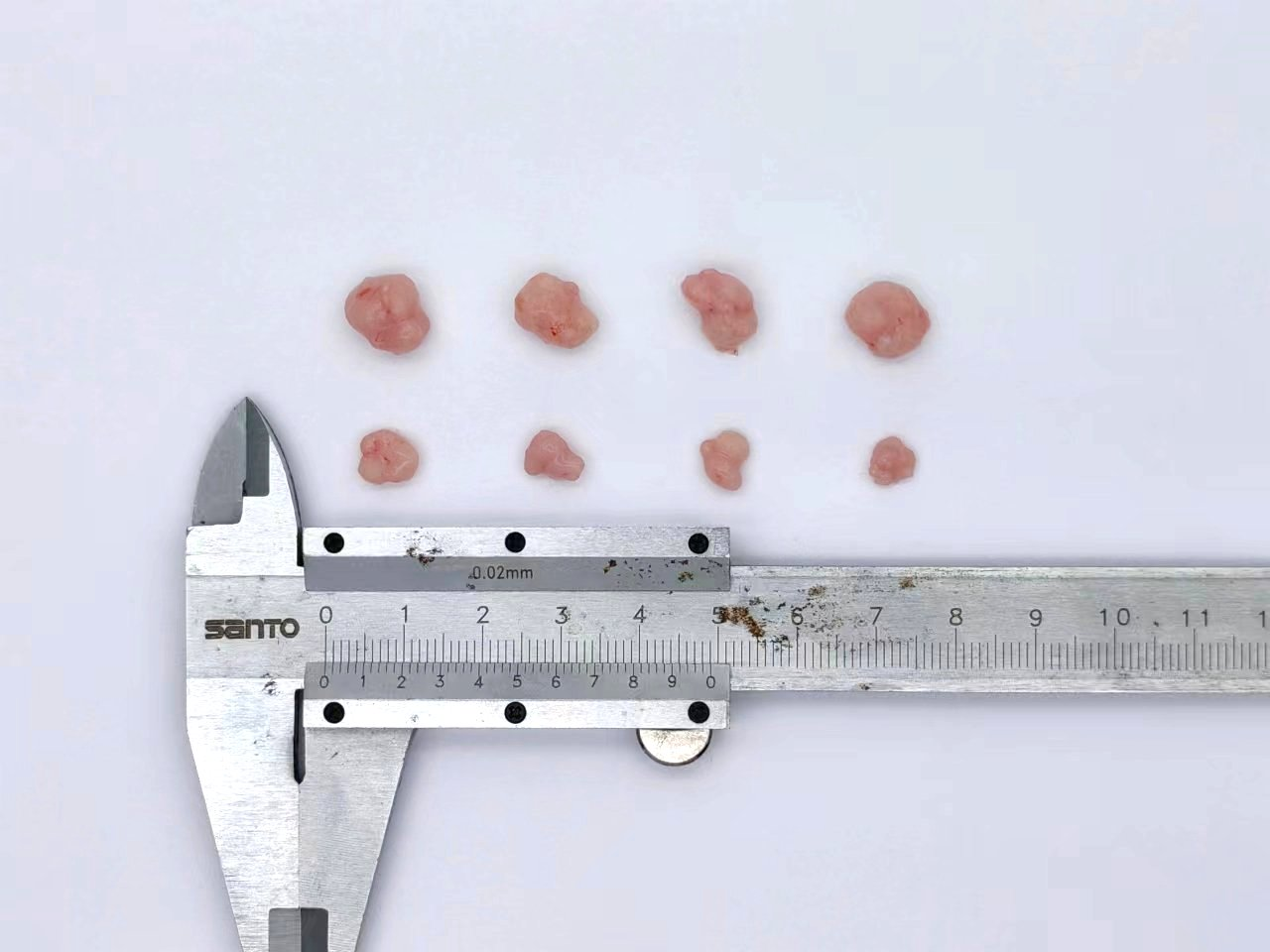

Supplement: Supplementary file 6 — Source data Fig. 5 [file 44319_2025_573_MOESM6_ESM.zip › Figure 5 Source Data/5B/Fig 5B-Xenograft tumor.tif]

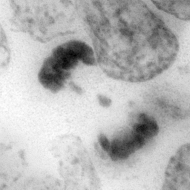

Supplement: Supplementary file 6 — Source data Fig. 5 [file 44319_2025_573_MOESM6_ESM.zip › Figure 5 Source Data/5C/LDHA KD-rLDHA T18A.tif]

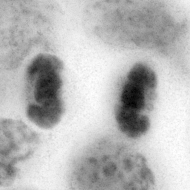

Supplement: Supplementary file 6 — Source data Fig. 5 [file 44319_2025_573_MOESM6_ESM.zip › Figure 5 Source Data/5C/LDHA KD-rLDHA WT.tif]

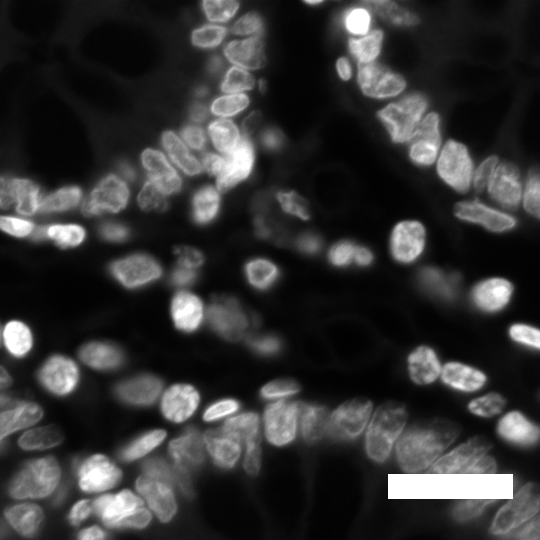

Supplement: Supplementary file 6 — Source data Fig. 5 [file 44319_2025_573_MOESM6_ESM.zip › Figure 5 Source Data/5D/paracancerous-DAPI.tif]

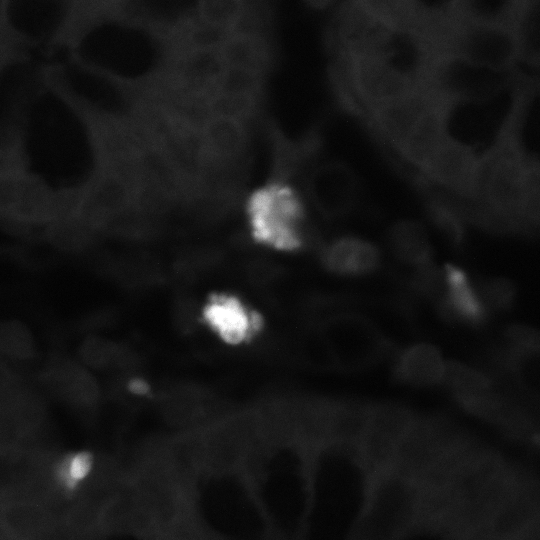

Supplement: Supplementary file 6 — Source data Fig. 5 [file 44319_2025_573_MOESM6_ESM.zip › Figure 5 Source Data/5D/paracancerous-H3 pS10.tif]

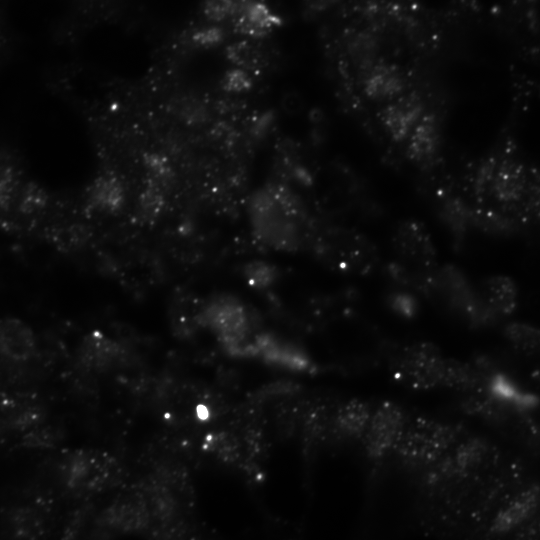

Supplement: Supplementary file 6 — Source data Fig. 5 [file 44319_2025_573_MOESM6_ESM.zip › Figure 5 Source Data/5D/paracancerous-LDHA pT18.tif]

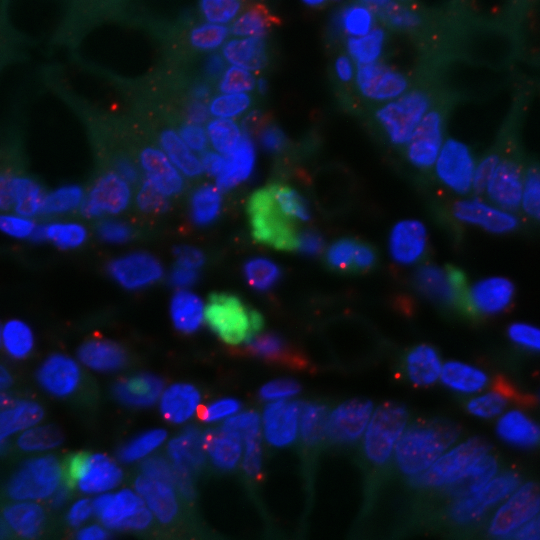

Supplement: Supplementary file 6 — Source data Fig. 5 [file 44319_2025_573_MOESM6_ESM.zip › Figure 5 Source Data/5D/paracancerous-Merge.tif]

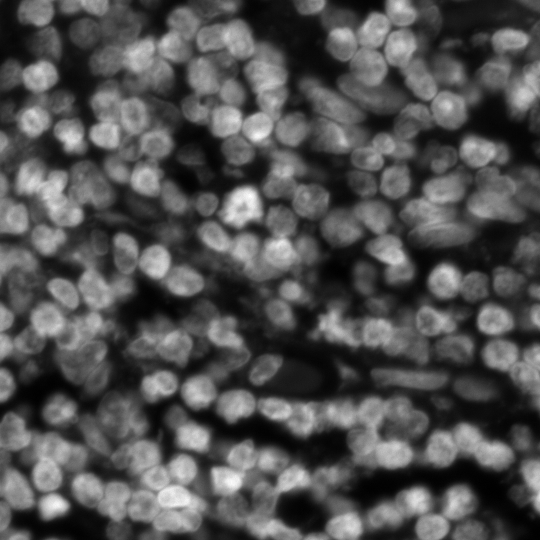

Supplement: Supplementary file 6 — Source data Fig. 5 [file 44319_2025_573_MOESM6_ESM.zip › Figure 5 Source Data/5D/Tumor-DAPI.tif]

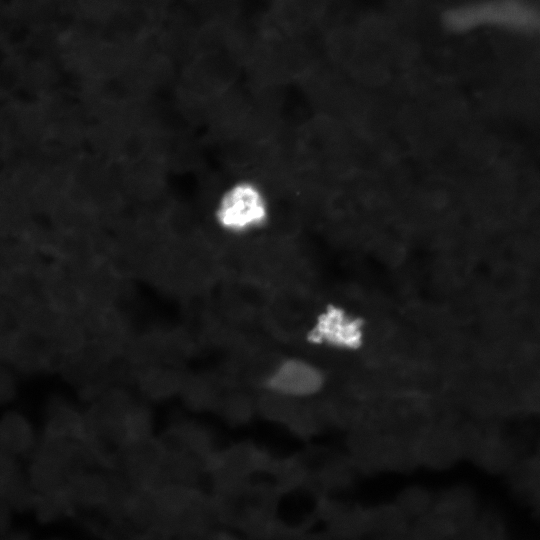

Supplement: Supplementary file 6 — Source data Fig. 5 [file 44319_2025_573_MOESM6_ESM.zip › Figure 5 Source Data/5D/Tumor-H3 pS10.tif]

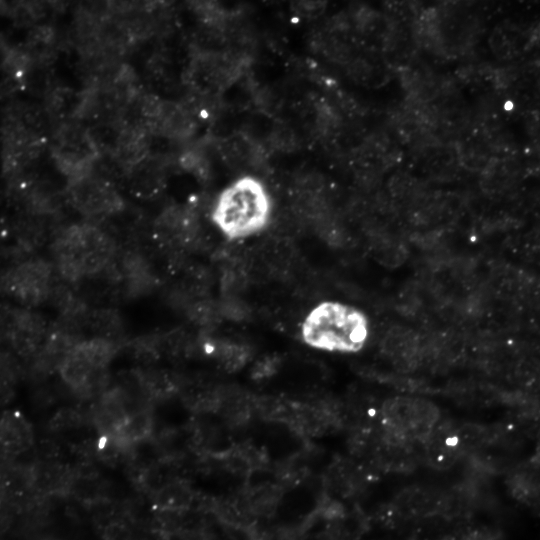

Supplement: Supplementary file 6 — Source data Fig. 5 [file 44319_2025_573_MOESM6_ESM.zip › Figure 5 Source Data/5D/Tumor-LDHA pT18.tif]

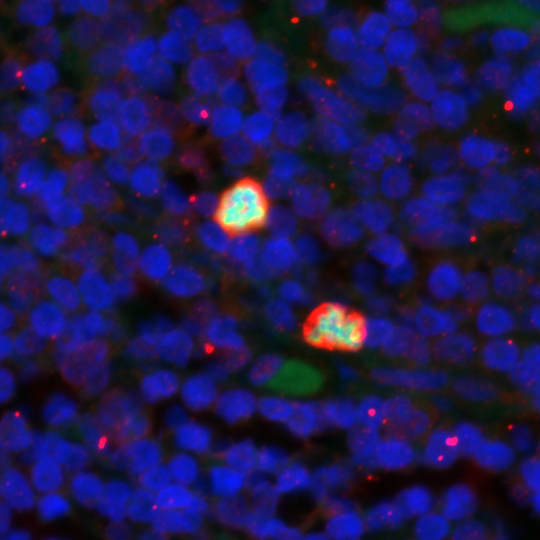

Supplement: Supplementary file 6 — Source data Fig. 5 [file 44319_2025_573_MOESM6_ESM.zip › Figure 5 Source Data/5D/Tumor-Merge.tif]

Fig. EV2D

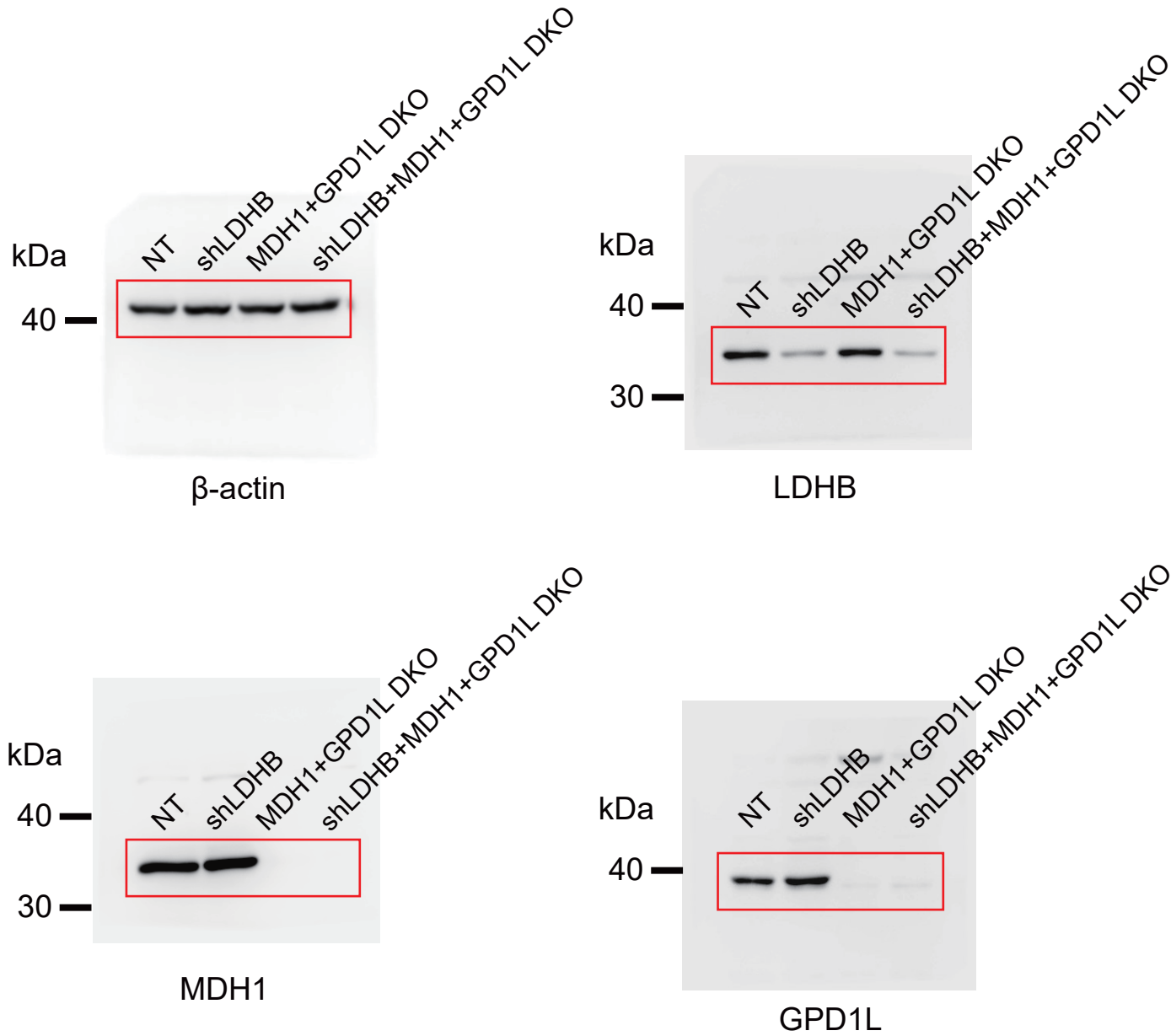

Supplement: Supplementary file 7 — Figure EVs Source Data [file 44319_2025_573_MOESM7_ESM.zip › Figure EV Source Data/EV2D/Fig. EV2D.pdf]
