# Supplementary material for: CDK1-mediated phosphorylation of LDHA fuels mitosis through LDHB-dependent lactate oxidation
Source: EMBO Rep. 2025 Sep 12;26(20):4923–49. doi: 10.1038/s44319-025-00573-8 (PMC12550033; doi:10.1038/s44319-025-00573-8)
Supplement: Supplementary file 5 — Source data Fig. 4 [file 44319_2025_573_MOESM5_ESM.zip › Figure 4 Source Data/4A/Fig.4A-Autoradiography.pdf]

Fig. 4A

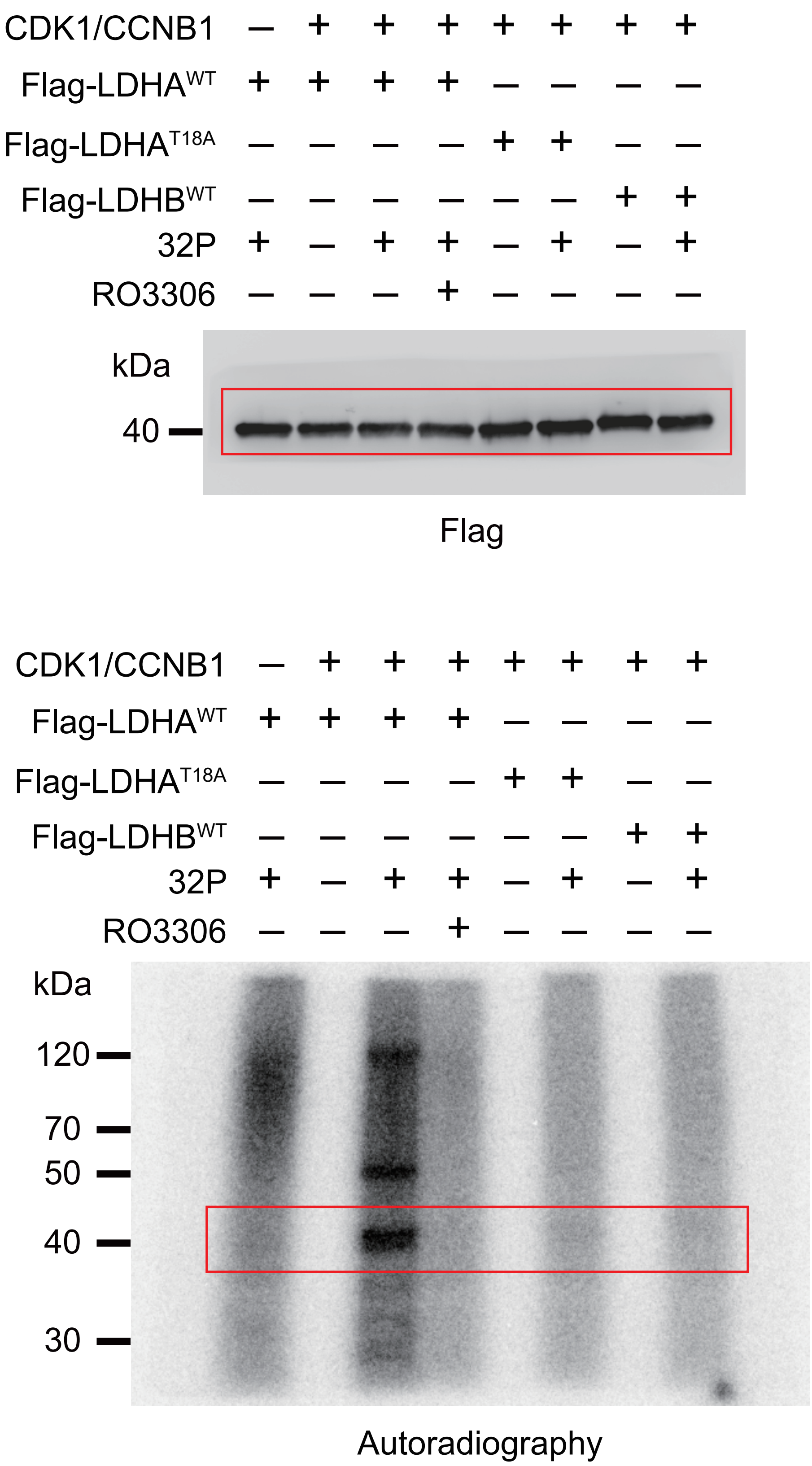
CDK1/CCNB1 – + + + + + + +
Flag-LDHA^{WT} + + + + – – – –
Flag-LDHA^{T18A} – – – – + + – –
Flag-LDHB^{WT} – – – – – – + +
32P + – + + – + – +
RO3306 – – – + – – – –
kDa
40
Flag
CDK1/CCNB1 – + + + + + + +
Flag-LDHA^{WT} + + + + – – – –
Flag-LDHA^{T18A} – – – – + + – –
Flag-LDHB^{WT} – – – – – – + +
32P + – + + – + – +
RO3306 – – – + – – – –
kDa
120
70
50
40
30
Autoradiography
