# Supplementary material for: CDK1-mediated phosphorylation of LDHA fuels mitosis through LDHB-dependent lactate oxidation
Source: EMBO Rep. 2025 Sep 12;26(20):4923–49. doi: 10.1038/s44319-025-00573-8 (PMC12550033; doi:10.1038/s44319-025-00573-8)
Supplement: Supplementary file 8 — Expanded View Figures [file 44319_2025_573_MOESM8_ESM.pdf]

# Expanded View Figures

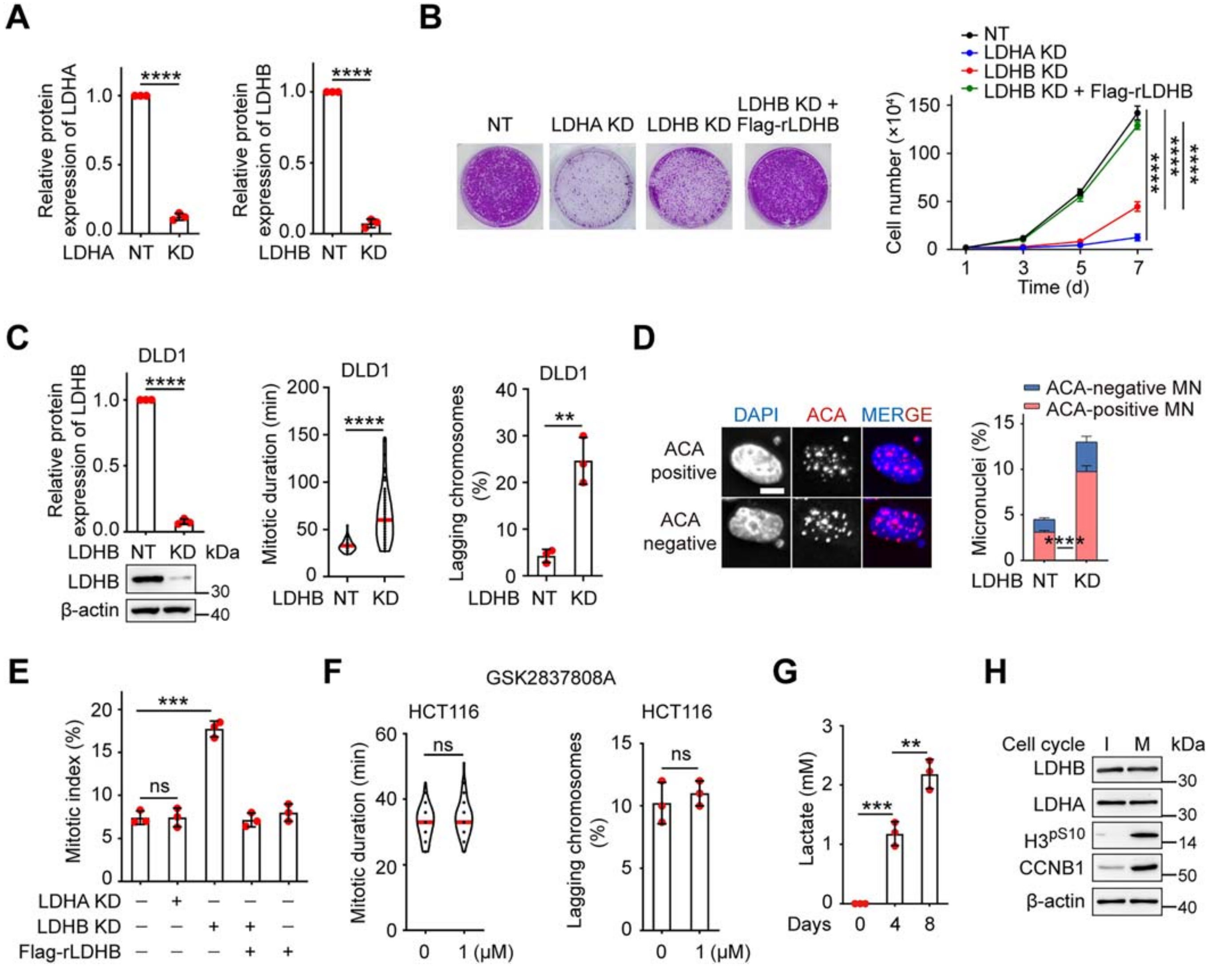


**Figure EV1. LDHB, not LDHA, is required for proper chromosome segregation.**

(**A**) The relative expression levels of LDHA and LDHB knockdown in Fig. 1A were analyzed ($n = 3$ biologically independent experiments; ****$P < 0.0001$). NT (non-targeting), KD (knockdown). (**B**) A colony formation assay was performed on the HeLa cells. After 10 days of culture, the cells were fixed and stained with crystal violet (left). A cell proliferation assay was conducted. Growth curves were plotted over a seven-day period ($n = 3$ biologically independent samples for each group; ****$P < 0.0001$) (right). (**C**) Cell lysates from DLD1 WT or LDHB KD cells were immunoblotted with the indicated antibodies, and the quantitative analysis is presented ($n = 3$ biologically independent experiments; ****$P < 0.0001$) (left). The mitotic duration was quantified ($n = 95$, 95 biologically independent cells; ****$P < 0.0001$), and quantification of lagging chromosomes is depicted ($n = 3$ biologically independent experiments; **$P = 0.0025$) (right). (**D**) HeLa WT and LDHB KD cells were stained with DAPI (blue) and anti-centromere antibodies (ACA) (red). Representative fluorescence images of centromere-positive and centromere-negative micronuclei are shown. Scale bar, 10 μm (left). The quantification of centromere-positive and centromere-negative micronuclei (MN) is presented ($n = 3$ biologically independent experiments; ****$P < 0.0001$) (right). (**E**) Cells were synchronized using a double thymidine block procedure and then fixed for DAPI staining 9 h after release. The mitotic index of each group was quantified ($n = 3$ biologically independent experiments; (ns) $P = 0.9679$, ***$P = 0.0001$). (**F**) HCT116 cells (H2B-GFP-labeled) treated with or without LDHA inhibitor GSK2837808A were monitored by time-lapse microscopy. The mitotic duration (min) starting from nuclear envelope breakdown to anaphase onset was quantified ($n = 71$, 71 biologically independent cells; (ns) $P = 0.0645$). Quantification of lagging chromosomes is shown ($n = 3$ biologically independent experiments; (ns) $P = 0.5313$). (**G**) Lactate levels in the culture medium of control organoids were measured at different culture time points using a lactate assay kit, and the medium was refreshed on day 4. ($n = 3$ biologically independent experiments; ***$P = 0.0005$, **$P = 0.0054$). (**H**) Western blot analysis was conducted to examine the expression of LDHA and LDHB in interphase and mitotic HeLa cells. Cyclin B1 (CCNB1) and $H3^{pS10}$ were utilized as mitotic markers. Immunoblots are representative of three independent experiments. Data Information: Data were shown as mean ± SD for (**A–C**) (left, right), (**D–F**) (right), (**G**); or as violin plots for (**C**) (middle), (**F**) (left). Statistical significance was assessed by two-way ANOVA for (**B**); other data were determined by unpaired two-tailed Student's *t*-test.

 © The Author(s)

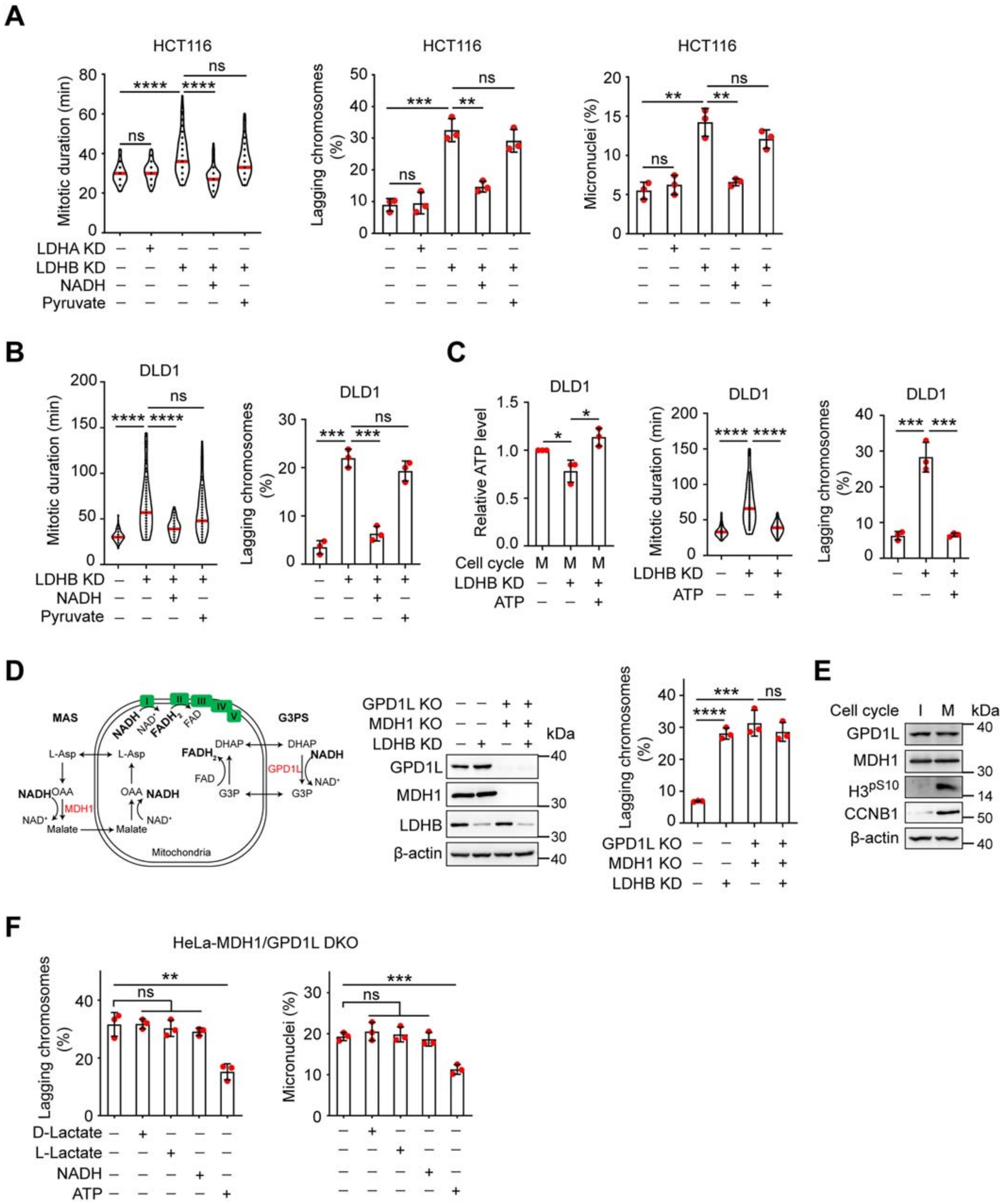
A
HCT116
Mitotic duration (min)
Lagging chromosomes (%)
Micronuclei (%)
LDHA KD
LDHB KD
NADH
Pyruvate
ns
B
DLD1
Mitotic duration (min)
Lagging chromosomes (%)
C
Relative ATP level
Cell cycle
ATP
D
MAS
G3PS
Mitochondria
GPD1L KO
MDH1 KO
GPD1L
MDH1
LDHB
β-actin
kDa
E
H3pS10
CCNB1
F
HeLa-MDH1/GPD1L DKO
D-Lactate
L-Lactate

**Figure EV2. The LDHB-mediated increase in mitotic NADH and ATP is essential for accurate chromosome segregation.**

(**A**) In HCT116-GFP-H2B cells, LDHA KD and LDHB KD cells treated with or without 1 mM NADH, 1 mM pyruvate were monitored using time-lapse microscopy. The mitotic duration was quantified ($n = 89, 89, 89, 89, 89$ biologically independent cells; (ns) $P = 0.0544$, ****$P < 0.0001$, (ns) $P = 0.0819$, from left to right) (left). The quantification of lagging chromosomes is presented ($n = 3$ biologically independent experiments; (ns) $P = 0.8262$, ***$P = 0.0006$, **$P = 0.0015$, (ns) $P = 0.3155$, from left to right) (middle). The quantification of micronuclei is shown ($n = 3$ biologically independent experiments; (ns) $P = 0.4795$, **$P = 0.0020$, **$P = 0.0020$, (ns) $P = 0.1592$, from left to right) (right). (**B**) In DLD1-GFP-H2B cells, LDHB KD cells were treated with 1 mM NADH or 1 mM pyruvate and monitored using time-lapse microscopy. The mitotic duration was quantified ($n = 116, 113, 100, 112$ biologically independent cells; ****$P < 0.0001$, (ns) $P = 0.0507$) (left). The quantification of lagging chromosomes is presented ($n = 3$ biologically independent experiments; ***$P = 0.0002$, ***$P = 0.0004$, (ns) $P = 0.1812$, from left to right) (right). (**C**) Relative changes of intracellular ATP levels in mitotic DLD1 cells upon LDHB KD or ATP supplementation were measured using an ATP assay kit ($n = 3$ biologically independent experiments; *$P = 0.0310$, *$P = 0.0142$) (left). Time-lapse microscopy was performed and mitotic duration was quantified ($n = 80, 80, 80$ biologically independent cells; ****$P < 0.0001$) (middle). The quantification of lagging chromosomes is presented ($n = 3$ biologically independent experiments; ***$P = 0.0009$, ***$P = 0.0008$, from left to right) (right). (**D**) A schematic illustration of two mitochondrial NADH shuttles, the malate-aspartate shuttle (MAS) and glycerol-3-phosphate shuttle (G3PS), is presented. The cytosolic enzymes malate dehydrogenase 1 (MDH1) and glycerol-3-phosphate dehydrogenase 1-like (GPD1L) are key components of MAS and G3PS, respectively (left). Extracts of HeLa WT, LDHB KD or MDH1 and GPD1L DKO cells were immunoblotted with the indicated antibodies (middle). Cells were filmed via time-lapse microscopy, and the quantification of lagging chromosomes is presented ($n = 3$ biologically independent experiments; ****$P < 0.0001$, ***$P = 0.0005$, (ns) $P = 0.4001$) (right). (**E**) Western blot analysis was conducted to examine the expression of MDH1 and GPD1L in interphase and mitotic HeLa cells. Cyclin B1 (CCNB1) and $H3^{pS10}$ were utilized as mitotic markers. (**F**) In Hela-GFP-H2B cells, MDH1 and GPD1L were knocked out and treated with 0.5 mM D-lactate, 0.5 mM L-lactate, 1 mM NADH, and 1 mM ATP. Cells were subsequently monitored using time-lapse microscopy. Quantification of lagging chromosomes and micronuclei is shown ($n = 3$ biologically independent experiments; (ns) $P = 0.9543$, (ns) $P = 0.6606$, (ns) $P = 0.3659$, **$P = 0.0046$, (ns) $P = 0.4387$, (ns) $P = 0.6970$, (ns) $P = 0.5850$, ***$P = 0.0009$, from left to right). Data Information: Data in **A** (left), **B** (left), and **C** (middle) are shown as violin plots; as mean ± SD for other data. Statistical significance was assessed by an unpaired two-tailed Student's *t*-test. Source data are available online for this figure.


© The Author(s)

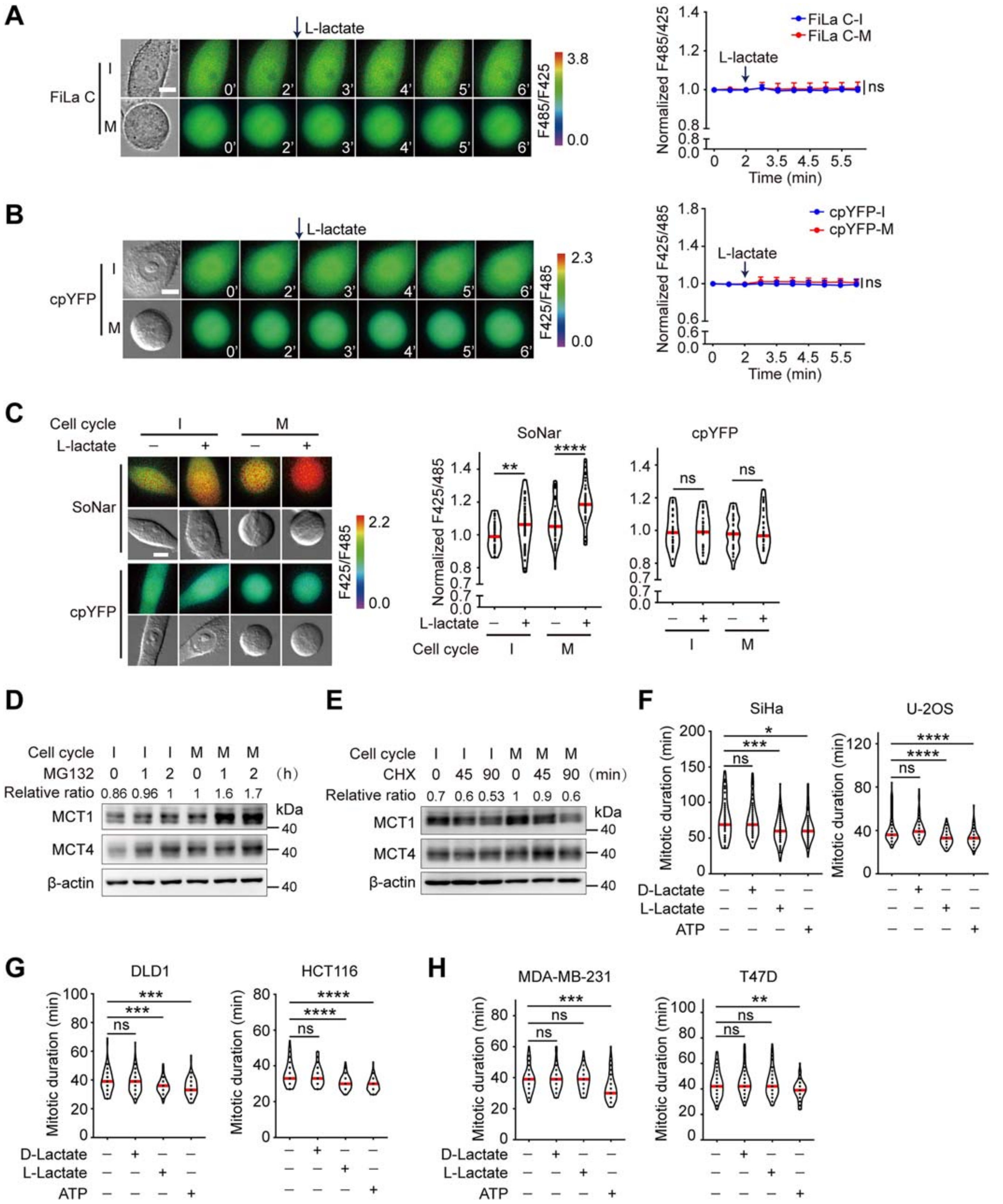
A
L-lactate
FiLa C
I
M
0' 2' 3' 4' 5' 6'
F485/F425
3.8
0.0
Normalized F485/425
FiLa C-I
FiLa C-M
L-lactate
ns
Time (min)
B
L-lactate
cpYFP
I
M
F425/F485
2.3
0.0
Normalized F425/485
cpYFP-I
cpYFP-M
L-lactate
ns
Time (min)
C
Cell cycle I M
L-lactate – + – +
SoNar
cpYFP
F425/F485
2.2
0.0
SoNar
cpYFP
Normalized F425/485
**
****
ns
ns
L-lactate
Cell cycle
D
Cell cycle I I I M M M
MG132 0 1 2 0 1 2 (h)
Relative ratio 0.86 0.96 1 1 1.6 1.7
MCT1
MCT4
β-actin
kDa
40
E
Cell cycle I I I M M M
CHX 0 45 90 0 45 90 (min)
Relative ratio 0.7 0.6 0.53 1 0.9 0.6
MCT1
MCT4
β-actin
kDa
40
F
SiHa
U-2OS
Mitotic duration (min)
D-Lactate
L-Lactate
ATP
G
DLD1
HCT116
Mitotic duration (min)
D-Lactate
L-Lactate
ATP
H
MDA-MB-231
T47D
Mitotic duration (min)
D-Lactate
L-Lactate
ATP

**Figure EV3. MCT1-mediated lactate import increases during mitosis to ensure accurate chromosome segregation.**

(**A**) Time-lapse microscopy was performed to monitor the relative changes of intracellular lactate levels using FiLa C (control) in interphase and mitotic HeLa cells, both before and after treatment with 10 mM lactate. Images were pseudo-colored based on the F485/F425 ratio. Scale bar: 10 µm (left). Data were quantified over time ($n = 10$ biologically independent cells for interphase and $n = 11$ for mitosis; (ns) $P = 0.8637$) (right). (**B**) Time-lapse microscopy was conducted to measure the relative changes of intracellular NADH/NAD$^+$ ratios using cpYFP (control) in interphase and mitotic HeLa cells, both before and after treatment with 10 mM lactate. Images were pseudo-colored based on the F425/F485 ratio. Scale bar: 10 µm (left). Data were quantified over time ($n = 9$ biologically independent cells for interphase and $n = 8$ for mitosis; (ns) $P = 0.3218$) (right). (**C**) The plasmid of NADH/NAD$^+$ sensor SoNar and control sensor cpYFP were transfected into HeLa cells. Cells were synchronized at interphase and mitosis using a double thymidine block procedure. Interphase and mitotic HeLa cells underwent live-cell imaging for 5–10 min in DMEM medium (Gibco, A1443001) supplemented with 10% FBS and 1% P/S. Subsequently, 10 mM lactate was added for an additional 5–10 min of imaging. Images were pseudo-colored based on the F425/F485 ratio. Scale bar: 10 µm (left). The relative intracellular NADH/NAD$^+$ ratios of each group are quantified ($n = 50$, 50, 50, 50 biologically independent cells for SoNar, $n = 34$, 34, 34, 34 biologically independent cells for cpYFP; **$P = 0.0083$, ****$P < 0.0001$, (ns) $P = 0.5419$, (ns) $P = 0.6498$, from left to right) (right). (**D**, **E**) Interphase and mitotic HeLa cells were obtained using a double thymidine block and shake-off. When cells progressed into interphase and mitosis, cells were treated with 10 µM MG132 or 100 µg/mL CHX for varying durations, followed by western blot analysis to examine the translational or protein stability regulation of MCT1 protein levels. (**F**) SiHa/U-2OS (H2B-GFP-labeled) cells treated with 0.5 mM D-lactate, 0.5 mM L-lactate, 1 mM ATP were monitored using time-lapse microscopy. The mitotic duration was quantified ($n = 69$, 69, 69, 69 biologically independent cells for SiHa cells and $n = 74$, 74, 85, 85 for U-2OS cells; (ns) $P = 0.7957$, ***$P = 0.0005$, *$P = 0.0125$, (ns) $P = 0.8086$, ****$P < 0.0001$, from left to right). (**G**) DLD1/HCT116-GFP-H2B cells treated with 0.5 mM D-lactate, 0.5 mM L-lactate, and 1 mM ATP were monitored using time-lapse microscopy. The mitotic duration was quantified ($n = 90$, 90, 90, 90 biologically independent cells for DLD1 cells and $n = 89$, 89, 89, 89 for HCT116 cells; (ns) $P = 0.5033$, ***$P = 0.0004$, ***$P = 0.0001$, (ns) $P = 0.3690$, ****$P < 0.0001$, from left to right). (**H**) MDA-MB-231/T47D (H2B-GFP-labeled) cells treated with 0.5 mM D-lactate, 0.5 mM L-lactate, 1 mM ATP were monitored using time-lapse microscopy. The mitotic duration was quantified ($n = 67$, 68, 68, 79 biologically independent cells for MDA-MB-231 cells and $n = 71$, 72, 72, 74 for T47D cells; (ns) $P = 0.7768$, (ns) $P = 0.8532$, ***$P = 0.0002$, (ns) $P = 0.5361$, (ns) $P = 0.7743$, **$P = 0.0029$, from left to right). Data Information: Data in (**C**, **F**–**H**) are shown as violin plots; as mean ± SD for (**A**, **B**). Statistical significance was assessed by two-way ANOVA for (**A**, **B**); by unpaired two-tailed Student's *t*-test for (**C**, **F**–**H**).

 © The Author(s)

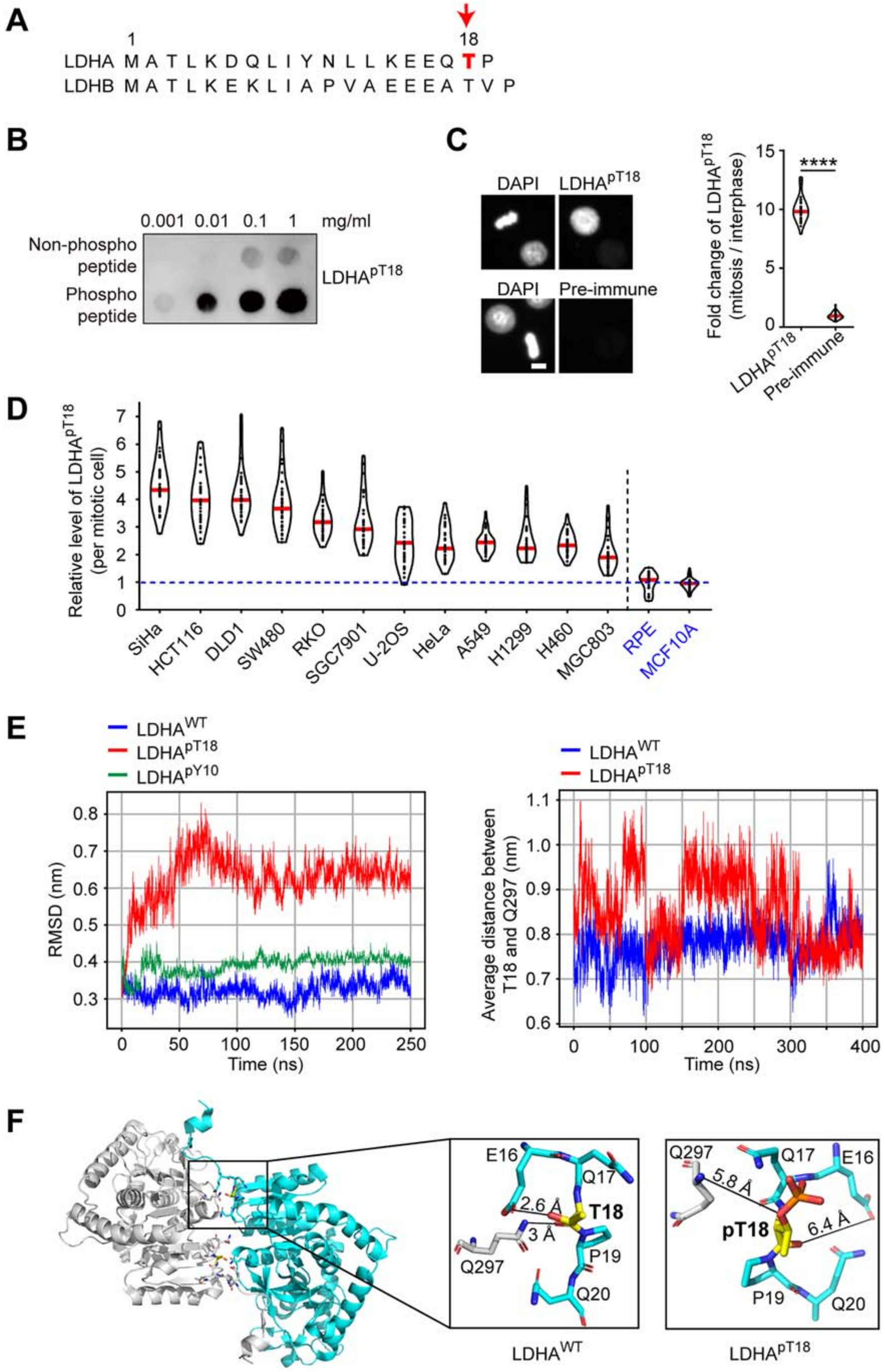
A
1
18
LDHA M A T L K D Q L I Y N L L K E E Q T P
LDHB M A T L K E K L I A P V A E E E A T V P
B
0.001 0.01 0.1 1 mg/ml
Non-phospho peptide
Phospho peptide
LDHApT18
C
DAPI
LDHApT18
DAPI
Pre-immune
Fold change of LDHApT18 (mitosis / interphase)
****
LDHApT18
Pre-immune
D
Relative level of LDHApT18 (per mitotic cell)
SiHa
HCT116
DLD1
SW480
RKO
SGC7901
U-2OS
HeLa
A549
H1299
H460
MGC803
RPE
MCF10A
E
LDHAWT
LDHApT18
LDHApY10
RMSD (nm)
Time (ns)
Average distance between T18 and Q297 (nm)
Time (ns)
F
E16
Q17
2.6 Å
T18
3 Å
Q297
P19
Q20
LDHAWT
Q297
5.8 Å
Q17
E16
pT18
6.4 Å
P19
Q20
LDHApT18

**Figure EV4. Phosphorylation of LDHA at T18 alters LDH tetramer formation during mitosis.**

(**A**) An alignment of the 1–20 amino acid sequences of LDHA and LDHB proteins is presented. (**B**) The $LDHA^{T18}$-phosphorylated peptide and non-phosphorylated peptide were added to the NC membrane, followed by a dot blot assay with anti-$LDHA^{pT18}$ antibody. (**C**) Representative images of interphase and mitotic HeLa cells stained with anti-$LDHA^{pT18}$ antibody or pre-immune antibody. Scale bar, 10 µm (left). Relative fold change of $LDHA^{pT18}$ (mitosis/interphase) is shown ($n = 43$, 39 biologically independent cells; ****$P < 0.0001$) (right). (**D**) Quantified mitotic $LDHA^{pT18}$ level across various cancer cell lines and two non-cancer cells are shown ($n = 34$, 34, 34, 32, 34, 34, 38, 32, 35, 33, 33, 34, 31, 35 biologically independent cells, from left to right). (**E**) The root-mean-square deviation (RMSD) of $LDHA^{WT}$, $LDHA^{pT18}$ and $LDHA^{pY10}$ was analyzed over a 250 ns timeframe during molecular dynamics simulations, with their RMSD values represented in blue, red, and green, respectively (left). Additionally, the distance between residue T18 or pT18 of LDHA and residue Q297 of another LDHA monomer was measured over a 400 ns timeframe during molecular dynamics simulations (right). (**F**) Molecular dynamics simulations revealed the dynamic conformational changes of LDHA T18 or pT18 and Q297 of another LDHA monomer in 70 ns. The residues Q297 and T18 or pT18 of LDHA were highlighted in color. Data Information: Data in (**C**, **D**) are shown as violin plots. Statistical significance was assessed by an unpaired two-tailed Student's *t*-test. Source data are available online for this figure.

 © The Author(s)

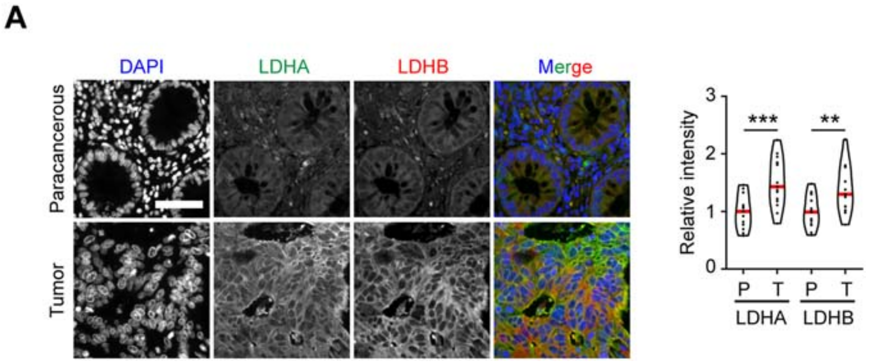


**Figure EV5. Phosphorylation of LDHA at T18 is essential for tumor progression.**

(**A**) Representative images of paracancerous and colorectal cancer (CRC) samples stained with DAPI (blue) and antibodies for LDHA (green) and LDHB (red). Scale bar, 50 μm (left). Quantification of relative expression levels of LDHA and LDHB in paired CRC samples is shown ($n = 15$ biologically independent samples; ***$P = 0.0007$, **$P = 0.0034$), P paracancerous, T tumor (right). Data Information: Data in (**A**) is shown as violin plots. Statistical significance was assessed by an unpaired two-tailed Student's *t*-test.
